# Fabrication and Characterization of Tetra-PEG-Derived Hydrogels of Controlled Softness

**DOI:** 10.1021/acs.macromol.5c00695

**Published:** 2025-06-25

**Authors:** Robert F Schmidt, Olga Matsarskaia, Takamasa Sakai, Michael Gradzielski

**Affiliations:** † 26524 Stranski-Laboratorium für Physikalische und Theoretische Chemie, Technische Universität Berlin, Strasse des 17. Juni 124, Berlin 10623, Germany; ‡ Institut Laue-Langevin, 71 Avenue des Martyrs, CS 20156, Grenoble, Cedex 9 38042, France; § Department of Chemistry & Biotechnology School of Engineering, 13143 The University of Tokyo, Tokyo 113-8656, Japan

## Abstract

Tetra-PEG hydrogels are known for their exceptionally homogeneous network structure and high mechanical stability. In this study, we demonstrate how the tetra-PEG framework can be adapted to create hydrogels of widely variable softness, with elastic moduli as low as 1–10 Pa. This is achieved by systematically tuning the ratio of tetra-functional and linear PEG macromers, producing networks with long, linear polymer segments that are intermittently cross-linked. The resulting hydrogels may serve as simple model systems for more complex biological hydrogels. Using small-angle neutron scattering (SANS), dynamic light scattering (DLS), rheometry, and microrheology, we reveal how the ratio of linear and tetra-functional precursor macromers influences the network structure and mechanical properties. These hydrogels, with precisely controllable rheological and structural characteristics, offer a versatile platform for studying the structure–property relationship in hydrogels mimicking the properties of biological systems such as mucus. The introduced principles are general and provide a foundation for designing new hydrogel materials with tailored properties for biomedical applications.

## Graphical abstract

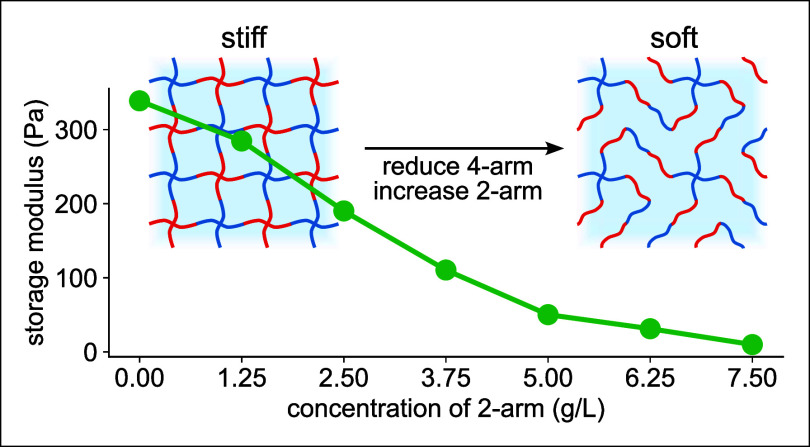

## Introduction

Tetra-PEG hydrogels are near-ideal polymer networks prepared from cross-end coupling of complementary tetra-functional poly­(ethylene glycol) (PEG) precursor polymers.^1–3^ They are known for their exceptionally homogeneous network structures, which are nearly defect-free, as demonstrated by small-angle neutron scattering (SANS),^4,5^ dynamic light scattering (DLS)^1^ or swelling^6^ experiments. This absence of defects leads to high mechanical strength at comparatively low polymer content.^7–9^ These qualities, coupled with their high biocompatibility, make tetra-PEG hydrogels highly appealing for biomedical applications, including drug delivery,^10–14^ bioadhesion/sealing^15,16^ and tissue engineering.^17–21^ Tetra-PEG hydrogels are also used as an artificial extracellular matrix. For example, Lust et al. studied the influence of the hydrogel stiffness on molecule diffusivity in tetra-PEG hydrogels designed to mimic the extracellular matrix.^22^

Most literature focuses on the advantages of using tetra-PEG hydrogels for the fabrication of tough hydrogels with elastic moduli in the kPa range,^1,7,16,17^ but softer gels can also be attractive for a number of potential applications. Through appropriate choice of the precursor macromers and their concentrations, it should also be possible to rationally design softer hydrogels using the tetra-PEG approach. Alternative strategies for modifying tetra-PEG-based hydrogels include dynamic covalent cross-linking approaches, such as hydrazone-linked tetra-PEG hydrogels (tetra-PEG DYNAgels) with high mechanical strength and remarkable self-healing properties.^23^

Soft hydrogels are also found abundantly in nature and in the human body. Examples include the extracellular matrix (ECM)^24^ and mucus, a very soft viscoelastic gel consisting of cross-linked glycoproteins called mucins with a storage modulus on the order of 1–10 Pa.^25–28^ Very soft tetra-PEG hydrogels, with finely tunable mechanical and structural properties, could thus serve as valuable model systems for these biological materials in future studies. Model systems, which are available in large quantity and reproducible quality, allow for systematic studies of structure–property relationships. Beyond hydrogels, several approaches have been reported for designing solvent-free, ultrasoft elastomers. Mpoukouvalas et al. synthesized soft elastomers by cross-linking 4-arm poly­(trimethylsilyloxyethyl acrylate) polymers and subsequently growing poly­(*n*-butyl acrylate) side chains from the network backbone, effectively embedding a covalently bound “solvent” within the structure.^29^ Maw et al. developed similarly soft materials using cross-linked bottlebrush polymers, where varying the length of side chains served to dilute and disentangle stress-supporting strands, leading to reduced stiffness.^30^ Additional strategies for the rational design of soft, tissue-mimicking polymeric networks have been comprehensively reviewed by Sheiko et al.^31^

The present paper explores the preparation, characterization and theoretical description of ultrasoft hydrogels drawing inspiration from the clear network formation principles employed in tetra-PEG hydrogels. The resulting hydrogels are comprehensively characterized using rotational rheometry, microrheology, DLS and SANS to establish structure–rheology relationships.

## Experimental Section

### Sample Preparation

To prepare very soft hydrogels based on the tetra-PEG framework, we employ a combination of tetrafunctional 20 kDa and linear bifunctional 10 kDa precursor PEG macromers, namely 4-arm and 2-arm PEG-thiol, as well as 4-arm and 2-arm PEG-maleimide. The two complementary species undergo a thiol-Michael addition click reaction.^9,32,33^ The precursor macromers are shown in Figure 1A.

**Figure 1:**
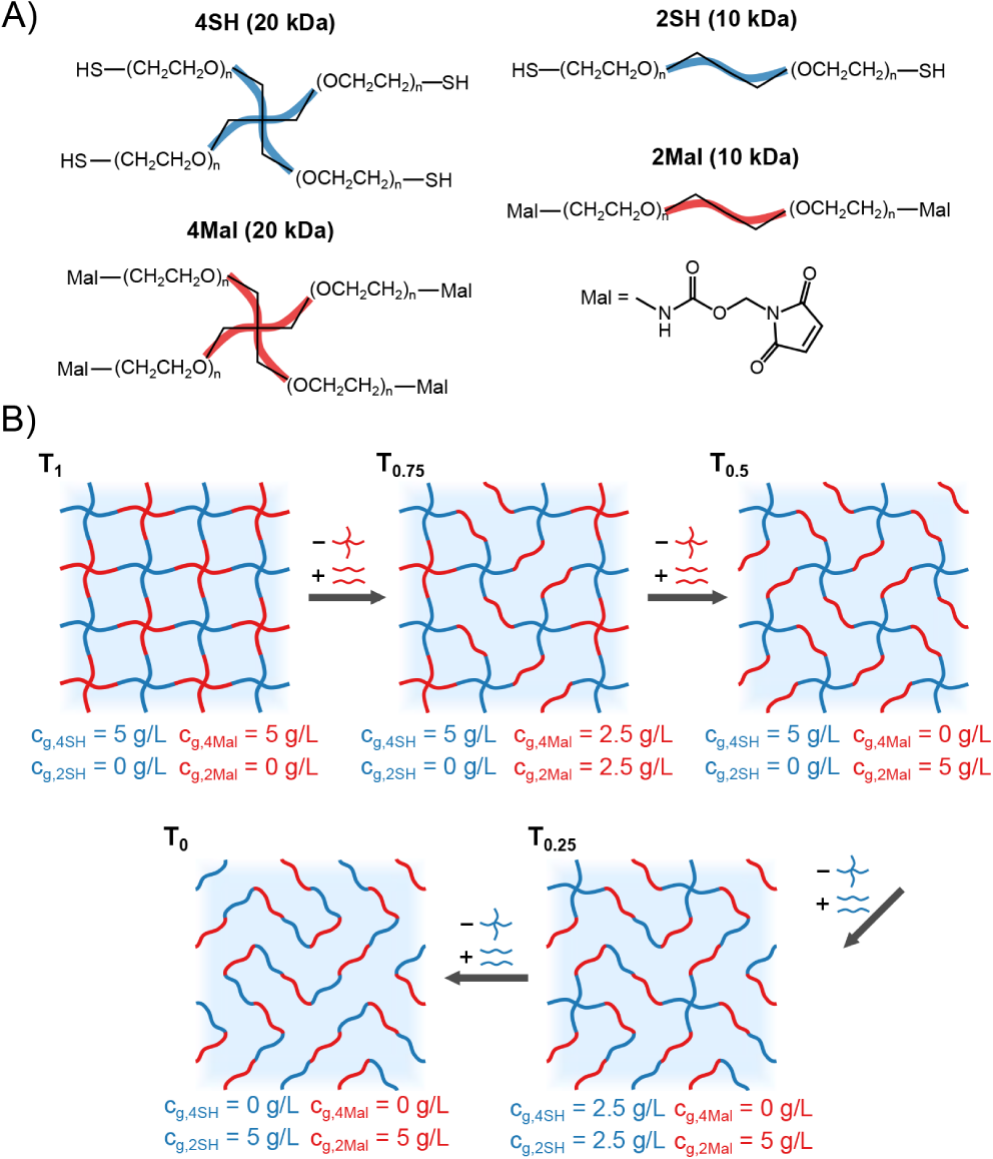
(A) The precursor molecules used for the preparation of soft tetra-PEG-derived hydrogels. (B) Simplified sketches of the supposed structures and compositions of a number of hydrogels. The concentration of 4-arm cross-linkers is continuously decreased, while simultaneously increasing the concentration of linear PEG molecules while keeping the overall polymer content constant at 10 g/L. The samples are labeled with T_*x*_, where *x* is the mass ratio of cross-linker (4Mal + 4SH) to the total mass of polymer.

We start from a standard 4 × 4 configuration, albeit at a low overall polymer concentration of 10 g/L. This concentration is significantly below the overlap concentration for 20 kDa 4-arm PEG-macromers, which is around 35 g/L.^4,34^ The overlap concentration for 10 kDa 2-arm PEG-macromers can be presumed to be similar, since the longest continuous segment in a 20 kDa 4-arm macromer is also 10 kDa. Preparing tetra-PEG gels at concentrations below the overlap concentration will generally lead to rather sparse polymer networks with a higher degree of structural defects,^35^ such as closed loops or dangling ends. This will result in softer hydrogels, which is desired for this project. The sketch of a defect-free network for T_1_ in Figure 1B is therefore idealized and meant only to illustrate the following procedure. Continuing from the 4 × 4 network, increasingly large fractions of the 4-armed PEG macromers are replaced by 2-arm PEGs, which should widen the mesh structure even further. Once all 4-arm PEGs have been replaced by 2-arm PEGs, there are no longer any cross-links, meaning the gel character is lost entirely. It is expected that at an intermediate composition, a structure consisting of sparsely interconnected linear segments will be formed. Furthermore, we impose the following conditions: first, the overall polymer content should remain constant at 10 g/L and second, the total number of thiol groups should be equal to the total number of maleimide groups. Given these two conditions, each 4-arm PEG should be replaced by two 2-arm PEG, where *M*_w_(4-arm) = 2*M*_w_(2-arm). Starting from a 4 × 4 tetra-PEG gel, prepared with 5 g/L 4-arm PEG-thiol (4SH) and 5 g/L 4-arm PEG-maleimide (4Mal), the 4Mal concentration is decreased in steps of 1.25 g/L, while simultaneously increasing the 2Mal concentration by the same amount until there is no 4Mal left. Afterward, the 4-arm PEG-thiol (4SH) concentration is successively decreased in steps of 1.25 g/L while simultaneously increasing the 2-arm PEG-thiol (2SH) concentration by the same amount until there is no 4SH left. This leads to a total of 9 samples, which shall hereafter be labeled as T_*x*_, where *x* is the mass fraction of cross-linkers (4SH + 4Mal) in the total mass of polymer. Sketches of the supposed structures of five of the hydrogels are shown in Figure 1B.

Notably, all T_*x*_ samples with *x* ≥ 0.25 remained intact without dissolving when submerged in water over several days, indicating the formation of stable, percolated networks, i.e., true gels rather than highly viscoelastic liquids. Due to the extreme softness of these gels, however, standard swelling experiments could not be performed reliably without disrupting and breaking the gel structure during handling. A video showing the soft, mucus-like behavior of sample T_0.25_ is given in the Supporting Information. Matsunaga et al. previously reported problems with determining swelling ratios of similar tetra-PEG gels prepared below the overlapping concentration.^4^ As described in the Supporting Information, we attempted to estimate the equilibrium swelling ratios by swelling pieces of gel inside a cell strainer. However, we could not obtain reliable or reproducible values, since some of the gels appeared to decrease in mass during swelling, which likely indicates problems with the swelling procedure rather than reflecting the true swelling behavior of the gels. Refer to the Supporting Information for further details.

### Experimental Procedures

More information about the experimental procedures and instrumental details are given in the Supporting Information.

## Results and Discussion

### Average Spacing

The most important structural parameter in a hydrogel is the mesh size, defined as the average distance between neighboring cross-links. However, this property is not experimentally accessible.^36^ In our system, the cross-links are provided by the 4-arm molecules.

To obtain a first estimate of the mesh size, we can look at the distribution of cross-links. In our system, the cross-links are provided by the 4-arm molecules. As a first approximation, we assume that all 4-arm molecules are evenly distributed in a cubic arrangement, that this distribution does not change during the reaction and that every 4-arm molecule becomes an effective cross-link. Under these assumptions, their average distance is given by

1 $$\xi_{\mathrm{calc}}={\left(c_{m}\left(4\mathrm{‐arm}\right)N_{A}\right)}^{-1/3}$$

where *c*_*m*_(4-arm) is the molar concentration of 4-arm molecules, and *N*_*A*_ is Avogadro’s constant. The resulting values for ξ_calc_ range from 14.9 nm for T_1_ to 29.8 nm for T_0.125_ as shown in Figure 2. In reality, not all 4-arm molecules will become effective cross-links due to structural defects, meaning the actual distance between cross-links should be larger than ξ_calc_.

**Figure 2:**
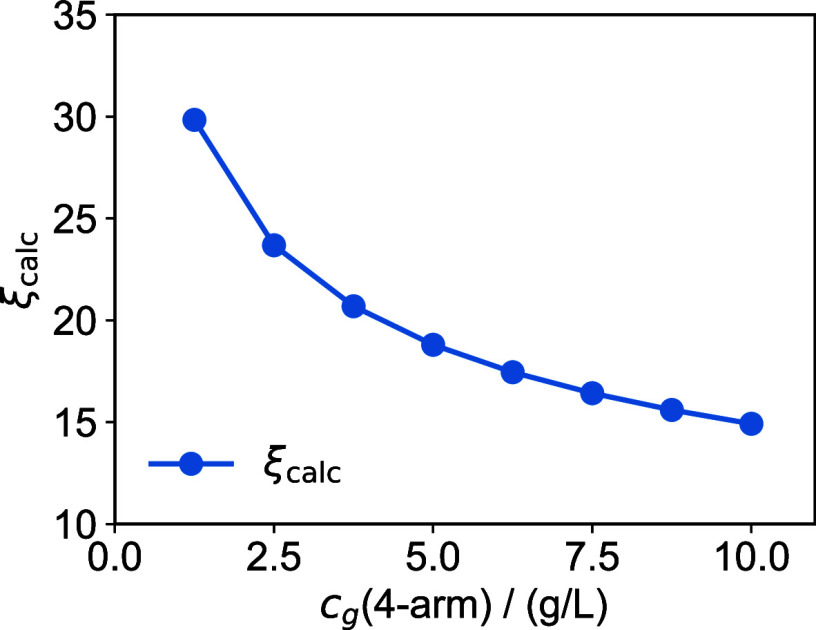
Average distance between 4-arm molecules, ξ_calc_.

### Conversion

To monitor the progress of the maleimide–thiol click reaction, UV/vis spectroscopy was employed. The unreacted maleimide species exhibits UV activity due to the π → π* transition of its C=C double bond, while the maleimide–thiol Michael addition product is UV inactive. Consequently, the absorbance of a solution containing maleimide and thiol species serves as a reliable indicator of reaction progress, as demonstrated in Figure 3.

**Figure 3:**
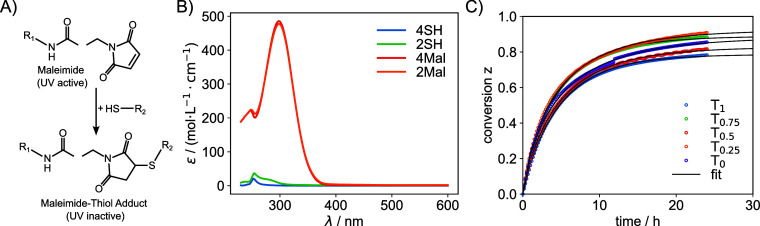
(A) During the maleimide–thiol reaction, the UV-active maleimide species is converted to the UV-inactive maleimide–thiol adduct. (B) Extinction spectra of the four precursor macromers (concentration always 10 g/L), where only the maleimide species exhibit UV activity. “mol” refers to the number of SH or Mal moieties. (C) Conversion *z* as a function of time fitted using eq 3.

Since the measured absorbance *A* is proportional to concentration according to Lambert–Beer’s law, the conversion of maleimide can be expressed as

2 $$z\left(t\right)=\frac{c_{m}\left(\mathrm{Mal‐S}\right)\left(t\right)}{c_{m,0}\left(\mathrm{Mal}\right)}=\frac{c_{m,0}\left(\mathrm{Mal}\right)-c_{m}\left(\mathrm{Mal}\right)\left(t\right)}{c_{m,0}\left(\mathrm{Mal}\right)}=1-\frac{A_{\mathrm{Mal},300\mathrm{nm}}\left(t\right)}{A_{\mathrm{Mal},300\mathrm{nm}}\left(0\right)}$$

where *c*_*m*_(Mal-S)­(*t*) and *c*_*m*_(Mal)­(*t*) represent the molar concentrations of the maleimide–thiol adduct and unreacted maleimide, respectively. *c*_*m*,0_(Mal) is the initial molar concentration of maleimide at *t* = 0, and *A*_Mal_,_300 nm_(*t*) and *A*_Mal_,_300 nm_(0) are the absorbances of the maleimide species at times *t* and *t* = 0, respectively. Since the SH species also absorb a small amount of light at λ = 300 nm, their contribution was subtracted (for details refer to the Supporting Information).

Figure 3C shows the conversion *z* as a function of time. The conversion increases steadily, and after 24 h, it approaches a plateau value of approximately 0.8, indicating that only 80% of the potential connections are formed. To accurately determine the asymptotic value of the conversion, *z*(*t* → ∞) = z_∞_, the data was fitted using the following function:

3 $$z\left(t\right)=z_{\infty }\left(1-\exp\left(-{\left(t/t_{z}\right)}^{a_{z}}\right)\right)$$

where *t*_*z*_ and *a*_*z*_ are fitting parameters. The fitted curves are shown as black lines in Figure 3C. The extracted values of *z*_∞_ are summarized in Table 1.

**Table 1:** Asymptotic Conversion Values (*z*_∞_) Obtained from Fitting eq 3

Sample	*z*_∞_
T_1_	0.80
T_0.75_	0.90
T_0.5_	0.84
T_0.25_	0.94
T_0_	0.90

The *z*_∞_ values exhibit significant variability and do not show any clear dependence on sample composition. Consequently, the average value of *z̅*_∞_ ≈ 0.86 is used for all other analyses.

### Rheology

The results of the macrorheological frequency sweep measurements (for experimental details, see Supporting Information) for samples T_1_ to T_0.25_ are shown in Figure 4. The remaining two samples, T_0.125_ and T_0_ displayed no gel-like characteristics, confirmed by the tube inversion method, and are referred to as sol samples. They could not be measured using this rheology setup. This indicates that *c*_*g*_(4SH + 4Mal) possesses a critical threshold concentration below which network formation does not occur.

**Figure 4:**
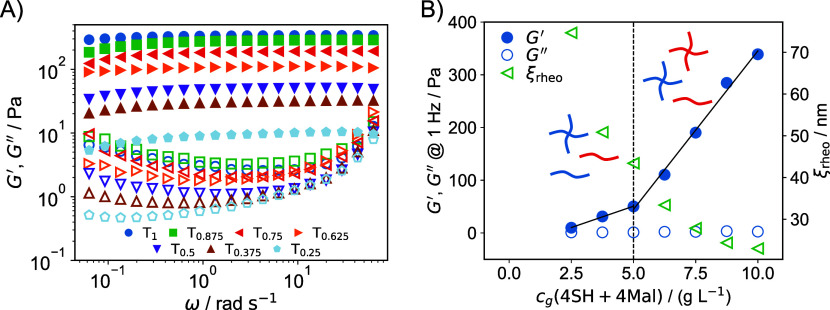
Results from macrorheology experiments. *G*′ and *G*″ are represented by solid and open symbols, respectively. (A) Frequency sweeps. (B) Values of *G*′ and *G*″ at a frequency of 1 Hz (6.28 rad/s) as a function of the cross-linker concentration. The curve can be divided into two linear sections, above and below 5 g/L, as indicated by the dashed black line and the linear fits (solid black lines). The available precursor macromers are shown for each section.

As shown in the frequency sweeps in Figure 4A, the modified tetra-PEG hydrogels exhibit predominantly elastic, gel-like behavior, with *G*′ > *G*″ across all samples and frequencies. By varying the composition, the storage modulus was reduced to approximately 10 Pa for T_0.25_. At even lower cross-linker concentrations, gel formation does not occur, and the samples exhibit nearly water-like viscosity, making them unsuitable for this rheometric setup.

For all samples, *G*′ exhibits minimal variation with frequency, though a slight decrease is noticeable at very low frequencies. This behavior indicates that all samples maintain a stable elastic network structure due to the presence of permanent chemical cross-links. Conversely, *G″* initially decreases slightly with frequency before rising again at higher frequencies. While no overall rearrangement of the elastic network occurs, slower dynamic processes, such as polymer chain fluctuations or rearrangements between cross-links, contribute to stress dissipation. It is important to note that based on the available frequency range, we cannot definitively rule out a potential crossover of *G*′ and *G*″ at lower frequencies, which would indicate very slowly relaxing viscoelastic liquids rather than true gels. However, the observation that all samples with *c*_*g*_(4-arm) ≥ 2.5 g/L remain intact and do not dissolve when submerged in water strongly supports the conclusion that these materials form stable gels, without a terminal flow regime at lower frequencies. The subsequent increase in *G*″ at higher frequencies could arise from the onset of Rouse modes of longer network segments. However, the relative magnitude of *G″* remains very small compared to *G*′. The loss tangents, shown in the Supporting Information, are all significantly below 1 and decrease with higher cross-linker content, indicating that the relative importance of the viscous properties are reduced.

Given the minimal frequency dependence of *G*′ and *G″*, their values at ω = 6.28 rad/s are plotted as a function of the cross-linker concentration (4SH + 4Mal) in Figure 4B. Visual inspection of Figure 4B reveals two distinct regions, separated at a cross-linker concentration of 5 g/L. In both regions, *G*′ increases approximately linearly, as indicated by the linear fits (solid black lines). However, a noticeable change in slope occurs above 5 g/L. This suggests that the elastic modulus is influenced not only by the total cross-linker concentration but also by the simultaneous presence of both types of cross-linkers (4SH and 4Mal), which apparently enhances the formation of an elastically effective network structure. The precursor macromers present in each region (excluding T_1_ and T_0_, which contain only two species) are shown to the left and right of the dashed vertical line in Figure 4B.

Two fundamental models for predicting the elastic modulus of a gel are the affine network model and the phantom network model. The affine network model assumes that each cross-link moves in direct proportion to the macroscopic deformation.^37^ The phantom network model additionally accounts for fluctuations around the average positions of the cross-linking points.^38^ The elastic modulus predictions for these models are expressed as follows:

4 $$G=\nu k_{B}T$$

and

5 $$G=\left(\nu -\mu \right)k_{B}T$$

respectively. Here, ν represents the number concentration of elastically effective network strands, μ denotes the number concentration of cross-links. Using eq 4 and assuming a cubic arrangement of the network strands, we can estimate a characteristic size (rheological blob size, related to the mesh size) according to

6 $$\xi_{\mathrm{rheo}}={\left(G/k_{b}T\right)}^{-1/3}$$

ξ_rheo_ is also shown in Figure 4B. It steadily decreases with the cross-linker concentration. The values of ξ_rheo_ are significantly larger than ξ_calc_, indicating that not all 4-arm molecules become elastically effective cross-links.

### Miller–Macosko Approximation

The simple network structure of tetra-PEG hydrogels makes them ideal candidates for testing theoretical predictions of hydrogel physical properties.

In a real hydrogel, not every 4-arm molecule necessarily acts as an elastically effective cross-link. The UV/vis experiments showed that only approximately 86% of the available maleimide groups undergo reaction. A 4-arm molecule becomes an effective cross-link only if three or four of its arms are connected to the network’s outer boundary. The Miller–Macosko (MM) tree-like theory provides a straightforward method to estimate the probability of a cross-link being elastically effective.^39^ This theory builds on Flory’s assumptions for an ideal network,^39,40^ which state that (i) all functional groups of the same type have equal reactivity, (ii) all groups behave independently, and (iii) no intramolecular reactions occur in finite species. While the MM theory provides a qualitative description of the network modulus, it does not account for structural defects such as closed loops or strand length polydispersity. Such features are inherent in real polymer networks and significantly impact the gel’s modulus.^41^ Recent work by Olsen et al.^41–43^ and Lang et al.^44,45^ explicitly incorporates such defects, resulting in more accurate theoretical predictions. In the following, we present a straightforward adaptation of the simple MM theory to our mixed 4-arm and 2-arm star polymer system. This serves primarily as an illustrative exercise, showing that the low moduli observed experimentally are also qualitatively consistent with theoretical predictions. A more rigorous theoretical treatment, following the approaches of Olsen et al. and Lang et al., could yield a more accurate description, but lies beyond the scope of the present work.

The MM analysis focuses on the probability that following one arm of a 4-arm molecule leads to a finite chain (a chain that does not connect to the sample’s edge). This probability is denoted as *P*(*F*_out_). In a system composed of two 4-arm species, A and B (in our case: 4SH and 4Mal), the probabilities *P*_*A*_(F_out_) and *P*_*B*_(*F*_out_) describe the likelihood that an arm of A or B, respectively, leads to a finite chain. Figure 5A illustrates this scenario.

**Figure 5:**
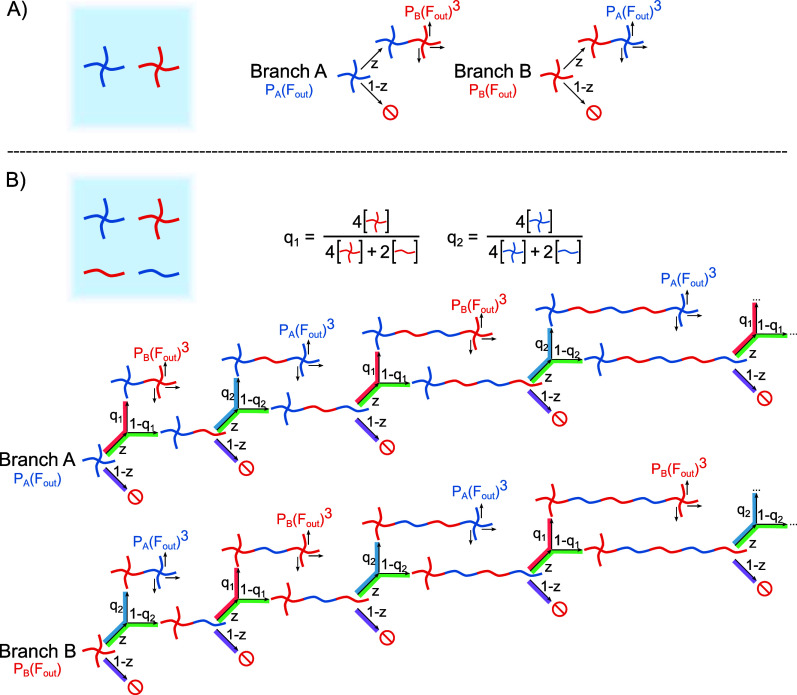
(A) Visualization of the Miller–Macosko theory for a system composed of two 4-arm molecules, A and B. (B) Visualization of the Miller–Macosko theory for a system consisting of two 4-arm molecules and two 2-arm molecules. There is an infinite number of possible paths, which can be divided into three groups. The stop paths are those which lead to a dangling chain (highlighted in purple). The linear path corresponds to the formation of an infinitely long linear chain (highlighted in green). The recursive paths lead back to A or B (highlighted in blue or red, respectively).

Starting at molecule A (blue) and following one of its arms, there are two possibilities: either the arm is connected to another molecule, or it is not. The probability of connection is given by the conversion *z* ≡ *z̅*_inf_, while the probability of no connection is 1 – *z*. If the arm is connected, the next molecule must be B (red). Thus, the overall probability that starting from A and following an arm leads to a finite chain is given by^46^

7 $$P_{A}\left(F_{\mathrm{out}}\right)=\left(1-z\right)+zP_{B}{\left(F_{\mathrm{out}}\right)}^{3}$$

Using the same reasoning, the probability for molecule B is

8 $$P_{B}\left(F_{\mathrm{out}}\right)=\left(1-z\right)+zP_{A}{\left(F_{\mathrm{out}}\right)}^{3}$$

Equations 7 and 8 form a set of coupled nonlinear equations. Under equimolar conditions, where *P*_*A*_(*F*_out_) = *P*_*B*_(*F*_out_), the system simplifies to a single equation, which can be solved analytically.

The modified tetra-PEG samples studied in this paper consist of four species (although only three are present at a time in a given sample): 4SH, 4Mal, 2SH, and 2Mal. Accordingly, the Miller–Macosko approximation is considerably more complex, as demonstrated in Figure 5B. Among the four precursor species, only 4SH and 4Mal function as cross-linkers, and thus only they need to be considered as starting points in the network analysis. A key distinction from the simpler scenario is that 4SH can now react with both 4Mal and 2Mal, introducing additional branching possibilities in the network. Consequently, the probabilities *P*_*A*_(*F*_out_) and *P*_*B*_(*F*_out_) are no longer equal and depend not only on the conversion *z* but also on the specific concentrations of 4Mal, 4SH, 2Mal, and 2SH. There is an infinite number of paths, which we divide into three groups. The stop paths are those which lead to a dangling chain (highlighted in purple in Figure 5B). The linear path corresponds to the formation of an infinitely long linear chain (highlighted in green). The recursive paths lead back to A or B (highlighted in blue or red, respectively). Despite this added complexity, *P*_*A*_(*F*_out_) and *P*_*B*_(*F*_out_) can still be calculated numerically, as detailed in the Supporting Information.

A 4-arm molecule becomes an effective cross-link if three or four of its arms are infinite. The probabilities for an A-4-arm molecule to have three or four infinite, elastically effective, arms are given by

9 PA(X3)=(43)PA(Fout)[1−PA(Fout)]3

and

10 PA(X4)=(44)[1−PA(Fout)]4

respectively. Analogous expressions hold for species B.

The concentrations of elastically effective cross-links and network strands can then be calculated as

11 $$\mu =c_{m}\left(A\right)N_{A}\cdot \left(P_{A}\left(X_{3}\right)+P_{A}\left(X_{4}\right)\right)+c_{m}\left(B\right)N_{A}\cdot \left(P_{B}\left(X_{3}\right)+P_{B}\left(X_{4}\right)\right)$$

and

12 $$\nu =c_{m}\left(A\right)N_{A}\left(\frac{3}{2}P_{A}\left(X_{3}\right)+\frac{4}{2}P_{A}\left(X_{4}\right)\right)+c_{m}\left(B\right)N_{A}\left(\frac{3}{2}P_{B}\left(X_{3}\right)+\frac{4}{2}P_{B}\left(X_{4}\right)\right)$$

respectively. Using these values, the elastic modulus can be computed with the affine and phantom network models (eqs 4 and 5). The comparison between these model predictions and experimental data is shown in Figure 6.

**Figure 6:**
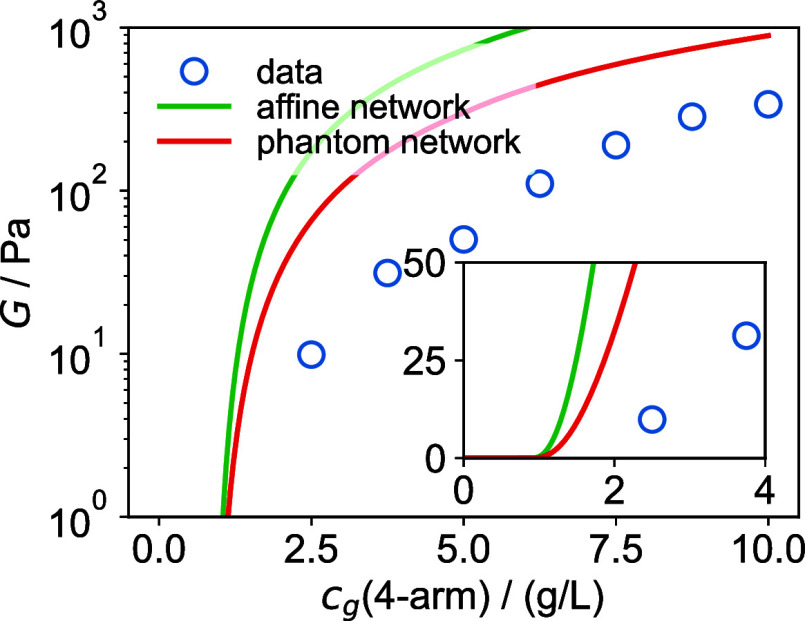
Comparison of the Miller–Macosko approximation predictions for the affine and phantom network models with experimental data (values of *G*′ at ω = 6.28 rad/s). The inset shows the onset region on a linear scale.

As shown in Figure 6, neither model accurately captures the absolute modulus values, but the phantom network model provides a closer approximation to the experimental data than the affine network model. This discrepancy is likely due to a high prevalence of closed loops, resulting from the low polymer concentrations used, which are well below the overlap concentration. Experimental studies have shown that ring formation is significantly more common in dilute systems,^47,48^ which may account for the greater deviations between Miller–Macosko predictions and experimental data observed here, as compared to the tetra-PEG hydrogels examined by Akagi et al.^46^ Additionally, more advanced theoretical treatments, following the procedures proposed by Olsen et al.^41–43^ and Lang et al.^44,45^ could provide a closer description. In our system, the ratio of the experimental shear modulus *G* to the phantom network prediction is approximately 0.3, suggesting that roughly 70% of the assumed elastically active cross-links are rendered ineffective due to loop formation. Representative examples of closed-loop structures likely present in this system are included in the Supporting Information. Nevertheless, the Miller–Macosko approximation does successfully predict a threshold concentration at which the network breaks down, albeit slightly shifted to lower concentrations compared with the data. The MM theory also allows to predict the sol fraction, i.e., the mass fraction of polymeric material, which is not connected to the infinite network and can be washed out if the gel is submerged in solvent. Its calculation is shown in the Supporting Information. The predicted sol fraction is 100% for *c*_*g*_(4-arm) < 1.0 g/L, drops to 15% for *c*_*g*_(4-arm) = 2.5 g/L and then quickly approaches 0 for *c*_*g*_(4-arm) > 5 g/L.

### Dynamic Light Scattering

The dynamics of the modified tetra-PEG hydrogels were analyzed using dynamic light scattering (DLS). Due to the chemical cross-linking in these hydrogels, the scattering elements are confined to fixed average positions. This behavior is termed nonergodic because the time-averaged correlation function differs from the ensemble-averaged correlation function.^49^ Consequently, the Siegert relation, which connects the intensity autocorrelation function, *g* ^(2)^(Δ*t*), to the field autocorrelation function, *g* ^(1)^(Δ*t*), is no longer valid.^49,50^ In a nonergodic system, the total scattered electric field is expressed as the sum of a constant component *E*_*C*_(q), arising from static inhomogeneities, and a fluctuating component *E*_*F*_(*q*,*t*), caused by thermal motion,^49,51,52^

13 $$E\left(q,t\right)=E_{F}\left(q,t\right)+E_{C}\left(q\right)$$

Here, *E*_*C*_(*q*) depends on the specific subensemble measured, whereas *E*_*F*_(*q*, *t*) does not. To appropriately analyze such samples, we adopted the nonergodic DLS framework proposed by Pusey.^49^ Under this approach, the field autocorrelation function is defined as

14 $$g^{\left(1\right)}\left(q,\Delta t\right)=1+\frac{1}{Y}\left[\sqrt{g^{\left(2\right)}\left(q,\Delta t\right)-\sigma_{I}^{2}}-1\right]$$

where

15 $$Y=\frac{\langle I\left(q\right)\rangle_{E}}{\langle I\left(q\right)\rangle_{T}}$$

and $\sigma_{I}^{2}=g^{\left(2\right)}\left(q,0\right)-1$ . The time-averaged scattering intensity, ⟨*I*(*q*)⟩_*T*_, corresponds to the intensity at one given position, while the ensemble-averaged intensity, ⟨*I*(*q*)⟩_*E*_, is derived by averaging over multiple sample positions (e.g., through continuous or stepwise rotation of the sample). The calculated *g* ^(1)^(*q*, Δ*t*) can then be analyzed in the normal fashion. The corresponding diffusion coefficient, *D*(*q*), represents the true diffusion coefficient and is generally smaller than the apparent diffusion coefficient that would be obtained by incorrectly treating a nonergodic sample as ergodic.^49^ Further experimental details are provided in the Supporting Information.

The results of the DLS measurements are presented in Figure 7. The field correlation functions, shown for a scattering angle of θ = 90°, decay to a finite plateau for the gel samples T_1_ to T_0.25_. This is due to the constant electric field component *E*_*C*_(*q*) in eq 13. These gel samples display a dominant relaxation mode at approximately 10^–4^ s. In contrast, the sol samples T_0.125_ and T_0_ decay to much lower values (the reason why they do not fully decay to zero is explained in the Supporting Information). Unlike the gel samples, the sol samples exhibit a second, significantly slower relaxation mode.

**Figure 7:**
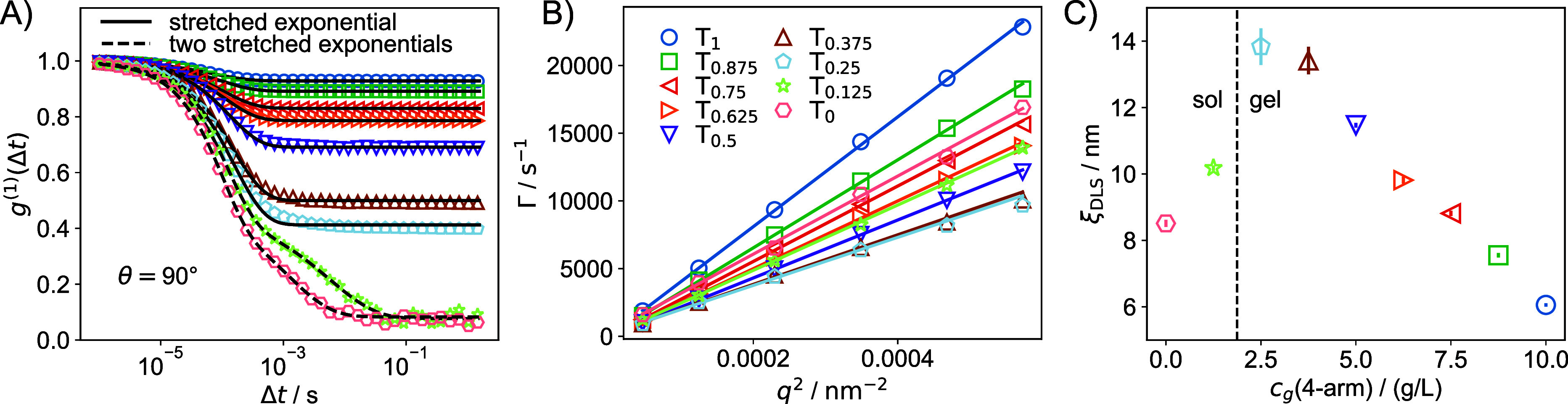
(A) Field autocorrelation functions determined using the nonergodic approach, shown for a scattering angle of θ = 90° (other angles are provided in the Supporting Information). The solid and broken black lines represent fits based on a stretched exponential function (eq 16) with *N* = 1 and *N* = 2, respectively. (B) The *q* ^2^-dependence of the relaxation rates, Γ, for the fast relaxation mode indicates diffusive behavior. (C) Characteristic size, ξ_DLS_, calculated using the Stokes–Einstein equation. The legend in (B) applies to all panels.

We fit *g* ^(1)^(Δ*t*) using a modified Kohlrausch–Williams–Watts stretched exponential model:

16 $$g^{\left(1\right)}\left(\Delta t\right)=\left(1-g_{\infty }\right)\cdot \left(\overset{N}{\underset{i=1}{\sum }}x_{i}\exp\left(-{\left(\Gamma_{i}\Delta t\right)}^{\beta_{i}}\right)\right)+g_{\infty }$$

where *N* is the number of relaxation modes (*N* = 1 for gel samples and *N* = 2 for sol samples), *x*_*i*_ is the strength of mode *i* (∑_i_ *x*_*i*_ = 1), β_*i*_ is the Kohlrausch stretching exponent, and *g*_∞_ is the value of g^(1)^(Δ*t*) as △*t* → ∞. The fitted results for gel and sol samples are displayed in Figure 7A as solid and broken black lines, respectively.

The relaxation rate, Γ, obtained from the fit of eq 16 to the *g* ^(1)^(Δ*t*) data, is the inverse of the characteristic relaxation time. For diffusive behavior, Γ = *Dq* ^2^, where *D* is the collective diffusion coefficient associated with the relaxation mode. Figure 7B shows that the gel mode is consistently diffusive. *D* was determined by linear fitting of Γ versus *q* ^2^ (solid lines in Figure 7B). The collective diffusion coefficient is related to the hydrodynamic radius, *R*_*h*_, using the Stokes–Einstein equation:

17 $$R_{h}=\frac{k_{B}T}{6\pi \eta D}$$

where η is the viscosity of water (0.89 mPa·s). The hydrodynamic radius was now related by us to a characteristic size, ξ_DLS_ of the polymer network,^53^ shown in Figure 7C. For the gel samples, ξ_DLS_ ranged from approximately 6 nm for T_1_ to 13 nm for T_0.25_.

Notably, our gel samples exhibited only one fast relaxation mode, associated with the cooperative diffusion of chains between neighboring cross-linking points. This contrasts with reports of a second, slower relaxation mode in similar systems, whose origin has been a topic of debate.^53,54^ In our DLS data, such a mode was absent for the gel samples T_1_–T_0.25_. Although ξ_DLS_ decreased appreciably for the sol samples, the values were still comparable, even for T_0_, which completely lacks cross-links. This aligns with the fact that DLS measures the dynamic correlation length, which is similar for polymer gels and polymer solutions of equivalent concentration.^5,53,55,56^ The slow relaxation mode observed in the sol samples showed no clear dependence on *q* and is therefore not further discussed here.

### Microrheology

Microrheology experiments were conducted by measuring the dynamic light scattering (DLS) of the hydrogels containing polystyrene tracer particles (diameter 192 nm). In DLS microrheology, the motion of embedded tracer particles is linked to the viscoelastic properties of the samples via the generalized Stokes–Einstein relation (GSER):^57–59^

$$G^{\prime }\left(\omega \right)=\left|G^{*}\left(\omega \right)\right|\cos\left[\pi \alpha \left(\omega \right)/2\right]$$

18 $$G^{\prime \prime }\left(\omega \right)=\left|G^{*}\left(\omega \right)\right|\sin\left[\pi \alpha \left(\omega \right)/2\right]$$

where

19 $$\left|G^{*}\left(\omega \right)\right|=\frac{k_{B}T}{\pi a\left\langle \Delta r^{2}\left(1/\omega \right)\right\rangle \Gamma \left[1+\alpha \left(\omega \right)\right]}$$

*a* being hydrodynamic radius of the tracer particles. The mean squared displacement (MSD) is directly related to the field autocorrelation function via:

20 $$g^{\left(1\right)}\left(t\right)=\exp\left(\frac{-q^{2}\left\langle \Delta r^{2}\left(t\right)\right\rangle }{6}\right)$$

The parameter α(ω) in eqs 18 and 19 is derived from a local power-law expansion of the MSD, $\left\langle \Delta r^{2}\left(t\right)\right\rangle \approx \left\langle \Delta r^{2}\left(1/\omega \right)\right\rangle {\left(\omega t\right)}^{\alpha \left(\omega \right)}$ .^59^ Details on the experimental procedure can be found in the Supporting Information. Results from the microrheology experiments are displayed in Figure 8.

**Figure 8:**
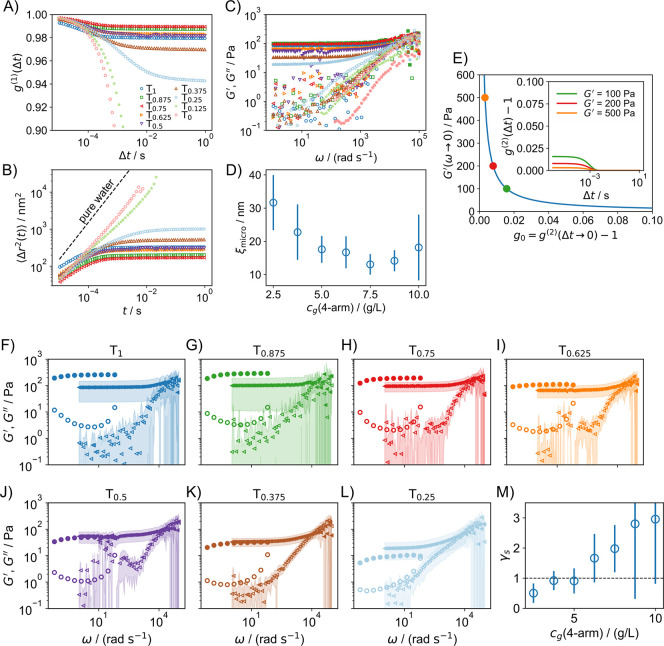
(A) Field autocorrelation functions. (B) Mean squared displacements (MSDs). (C) Microrheological viscoelastic moduli. (D) Characteristic size ξ_micro_ determined from the MSD plateau, $\xi_{\mathrm{micro}}=\sqrt{\left\langle \Delta r^{2}\left(t\to \infty \right)\right\rangle }$ . (E) For stiff hydrogels, *G*′ is directly related to the intercept of the intensity autocorrelation function. Large *G*′ values correspond to small intercepts. The inset shows hypothetical correlation functions for *G*′ values of 100, 200, and 500 Pa, modeled as simple exponential decays with a time constant of 1 ms. (F–L) Comparison of macrorheology (circles) and microrheology (triangles), with filled symbols denoting *G*′ and open symbols denoting *G″*. The data density was reduced by a factor of 2 for clarity. (M) Shift factor $\gamma_{s}={\left|G^{*}\right|}_{\mathrm{macro}}/{\left|G^{*}\right|}_{\mathrm{micro}}$ , with the dashed black line representing perfect agreement. The legend in (A) applies to (B,C) as well.

In Figure 8A, the field correlation functions are presented. For the gel samples, they decay to values of *g*_∞_ > 0.94, and for T_1_–T_0.5_, to *g*_∞_ > 0.98, highlighting their highly nonergodic behavior. Differences in *g*_∞_ values are small and negligible relative to the errors, which are omitted for clarity but available in the Supporting Information. For the sol samples (T_0.125_ and T_0_), *g* ^(1)^(Δ*t*) decays to zero, indicating ergodic behavior (not visible in A due to the *y*-axis scale). Figure 8B shows the MSDs derived using eq 20. For gel samples, the MSD approaches a plateau, $\left\langle \Delta r^{2}\left(t\to \infty \right)\right\rangle$ , indicating particle confinement by cross-links. The square root of the plateau MSD defines a characteristic size, $\xi_{\mathrm{micro}}=\sqrt{\left\langle \Delta r^{2}\left(t\to \infty \right)\right\rangle }$ , shown in Figure 8D that describes how far effectively a mesh point can move within the gel network. ξ_micro_ generally increases with decreasing cross-linker concentration, though the upturn for T_1_ and T_0.875_ likely reflects large errors rather than true trends. For the sol samples, the MSDs increase almost linearly, resembling the behavior of tracer particles in pure water (dashed line in Figure 8B).

Figure 8C illustrates the microrheological storage and loss moduli. For gel samples, *G*′ plateaus at low frequencies. *G* ^″^ is significantly smaller and the values scatter considerably. As per eqs 1 and 19, $\left|G^{*}\left(\omega \right)\right|$ is inversely proportional to the MSD. The magnitudes of *G*′ and *G″* depend on the gradient α­(ω). For gel samples, α­(ω) approaches zero in the MSD plateau region, making *G*′ ≫ *G*″. As a result, reliable values of *G*″ cannot be determined.

The *G*′ plateau values for T_1_–T_0.75_ are similar, indicating the difficulty of distinguishing moduli above ∼100 Pa using DLS microrheology under these experimental conditions. Using eqs 19, 20, and 14, it is easy to verify that for the gel samples (*G*′ ≫ *G*″), *G*′ can be approximated using

21 $$G^{\prime }\approx \left|G^{*}\right|\approx -\frac{k_{B}Tq^{2}}{6\pi a\ln g_{\infty }}=-\frac{k_{B}Tq^{2}}{3\pi a\ln\left(1-g_{0}\right)}$$

In the last equality, we have used that *Y* in eq 14 is equal to 1 if averaged for a large number of individual measurements. Using eq 21, *G*′ can be determined as a function of *g*_0_, as illustrated in Figure 8E. Achieving high values of *G*′ requires *g*_0_ to become very small. This is exemplified by hypothetical intensity correlation functions shown in the inset of Figure 8E. For instance, a *G*′ value of 500 Pa would necessitate the corresponding correlation function to decay from 0.003 to 0. Such extremely low intercepts are challenging to measure experimentally, which explains the difficulty in accurately characterizing stiff hydrogels using microrheology. According to eq 21, measurement precision could be improved by either increasing the scattering angle or reducing the tracer particle radius, as these adjustments would result in a higher intercept value.

Figure 8F–L compares microrheology and macrorheology from the same samples. While qualitative agreement is good, discrepancies exist, particularly for stiffer hydrogels where microrheology underestimates moduli. For weaker hydrogels, agreement improves, with microrheology exceeding macrorheology for T_0.25_. To quantify deviations, a shift factor $\gamma_{s}={\left|G^{*}\right|}_{\mathrm{macro}}/{\left|G^{*}\right|}_{\mathrm{micro}}$ was determined (Figure 8M). γ_*s*_ decreases from ∼3 for T_1_ to ∼0.5 for T_0.25_. These deviations likely stem from the inaccuracies in determining *g*_0_, as explained above.

## Small-Angle Neutron Scattering

To investigate the mesoscopic structure, SANS measurements were conducted on the modified tetra-PEG hydrogels. Details of the experimental procedure are provided in the Supporting Information. The SANS intensities on absolute scale, with background subtraction applied (hereafter referred to as *I*(*q*)), are displayed in Figure 9A. For polymer chains, the scattering intensity typically exhibits a scaling behavior proportional to ∼*q* ^–2^. To highlight subtle differences among similar spectra, *I*(*q*)·*q* ^2^ is plotted, yielding what are commonly known as Kratky plots.^60^ These Kratky plots are shown in Figure 9B.

**Figure 9:**
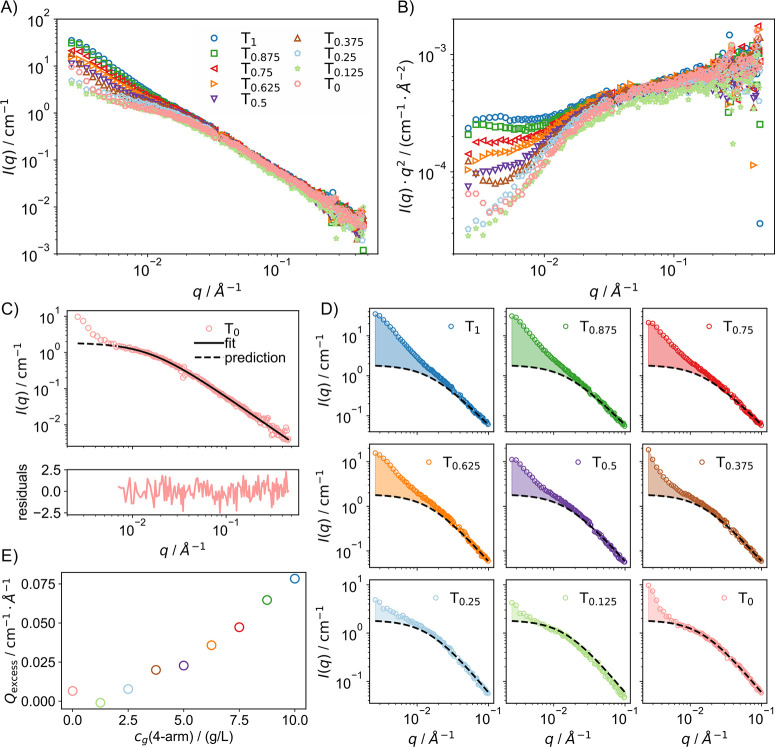
(A) SANS intensities with subtracted background for modified tetra-PEG hydrogels. (B) Kratky plots. (C) T_0_ fitted with the Ornstein–Zernike scattering function, eq 22, for *q* > 7 × 10^–3^ Å^–1^. (D) Assuming the Ornstein–Zernike scattering term remains identical for all samples (due to the constant polymer concentration), the fit function derived in (C) is overlaid on the spectra of all samples. The shaded area represents the excess scattering, *Q*_excess_. (E) The excess scattering increases with rising cross-linker concentration, indicating a stronger influence of large-scale inhomogeneities.

The SANS spectra show remarkable similarity in the mid- to high-*q* range, suggesting that the local structure is very similar across all samples. At low *q*, the intensity exhibits an upturn, which is most pronounced for T_1_ and progressively diminishes as the cross-linker concentration decreases. This low-*q* intensity rise indicates the presence of large-scale inhomogeneities, a characteristic feature frequently observed in polymer gels.^61,62^ Very interesting is certainly the observation that the intensity at low *q* increases with increasing cross-linker concentration. Normally one would expect a more and more homogeneous polymer network with increasing cross-linker concentration but apparently the opposite is the case here. Previous SANS studies on simple tetra-PEG hydrogels revealed a clear absence of such large-scale inhomogeneities, as evidenced by the lack of a low *q* intensity increase in the scattering spectra.^4,5^ An exception was observed in tetra-PEG gels made from very short, 5 kDa 4-arm precursor macromers.^5^ The pronounced inhomogeneities in our modified tetra-PEG gels likely stem from the low concentration of 10 g/L, which is below the overlap concentration. As discussed above, low polymer concentrations increase the likelihood of forming closed loops, which could account for the observed inhomogeneities at low *q*. A recent study on disulfide-cross-linked tetra-PEG hydrogels similarly showed a higher prevalence of structural defects for lower macromer concentrations.^63^

For *q* > 3 × 10^–2^ Å^–1^, the SANS spectra are nearly indistinguishable. According to de Gennes, the scattering behavior of an ideal polymer gel should be identical to that of a corresponding polymer solution at the same concentration.^64^ The scattering intensity of polymer chains can be described using the Ornstein–Zernike function:

22 $$I\left(q\right)=\frac{I\left(0\right)}{1+{\left(q\xi_{\mathrm{SANS}}\right)}^{m}}$$

where *I*(0) represents the intensity for *q* → 0, and ξ_SANS_ is the correlation length. The exponent *m* is inversely related to the Flory exponent, *m* = 1/ν. Specifically, *m* = 2 for polymers in theta solvents (ν = 0.5) and *m* ≈ 1.7 for polymers in good solvents (ν ≈ 0.588).^65^ The scattering of hydrogels with large-scale inhomogeneities is usually well described by the Hammouda model, consisting of the Ornstein–Zernike term, eq 22, and an additional term describing the Porod scattering of the inhomogeneities.^66,67^ However, as shown in the Supporting Information, the parameters in the Hammouda model cannot be independently determined from a fit of the data, due to a lack of distinctive features in the SANS spectra. This is particularly evident for the stiffer hydrogels, which appear almost as straight lines in the double logarithmic plot. This suggests that the samples have the same fractal dimension across the entire length scale probed by the SANS experiment. Instead of fitting a model, we will therefore quantify the contribution of the large-scale inhomogeneities by looking at the difference between the measured intensities and the Ornstein–Zernike term.

In the case of our modified tetra-PEG gels, the polymer concentration is constant at 10 g/L across all samples, with only the polymer species ratio varying. Thus, from the perspective of a polymer chain, the probability of encountering another chain at a given distance remains constant. Consequently, both ξ_SANS_ and *I*(0) should remain unchanged across all samples, explaining the lack of a marked difference in the SANS spectra between the gel and sol samples. To quantify the effect of inhomogeneities, the values of ξ_SANS_ and *I*(0) must first be determined. The plateau value *I*(0) is most apparent in the T_0_ sample, where the influence of large-scale inhomogeneities is minimal. Therefore, the T_0_ curve is fitted using eq 22, as shown in Figure 9C, with the fit range restricted to *q* > 7 × 10^–3^ Å^–1^, describing accurately the plateau.

Assuming that the Ornstein–Zernike scattering term is identical for all samples, the deviation between the measured spectra and the Ornstein–Zernike fit for T_0_ quantifies the influence of large-scale inhomogeneities. This deviation is quantified by

23 $$Q_{\mathrm{excess}}=\int_{q_{\mathrm{start}}}^{q_{\mathrm{end}}}\left(I_{\mathrm{sample}}\left(q\right)-I_{\mathrm{OZ}}\left(q\right)\right)dq$$

where *q*_start_ and *q*_end_ are the first and last *q* values, *I*_sample_ is the scattering intensity of the sample, and *I*_OZ_ is the Ornstein–Zernike fit result from T_0_. *Q*_excess_ represents the area between the measured data and the Ornstein–Zernike term, as illustrated in Figure 9D.

The excess scattering, Q_excess_, increases approximately linearly with the cross-linker concentration, as shown in Figure 9E. Despite the introduction of additional linear precursor species, which might complicate the potential structures, the influence of large-scale heterogeneities diminishes as the cross-linker content decreases. Although the simple 4 × 4 tetra-PEG architecture is expected to exhibit homogeneity on a local scale, at our rather low polymer concentration, cross-links appear to cluster in specific regions. This results in areas of high cross-linking density with significant scattering material, interspersed with regions that are relatively devoid of cross-links and polymer.

## Comparison of Characteristic Sizes

The modified tetra-PEG hydrogels were comprehensively characterized using a variety of experimental techniques, each yielding a characteristic size, ξ. These sizes are summarized below and shown in Figure 10.

- ξ_calc_: Average distance between 4-arm molecules, assuming cubic spacing.
- ξ_rheo_: Characteristic size derived from the storage modulus.
- ξ_MM_: Mesh size calculated from the concentration of elastically effective cross-links (μ) using the Miller–Macosko approximation.
- ξ_DLS_: Characteristic size determined via DLS using the nonergodic approach.
- ξ_micro_: Square root of the plateau value of the MSD for *t* → ∞, obtained from DLS microrheology.
- ξ_SANS_: Correlation length from the Ornstein–Zernike scattering function (eq 22) applied to T_0_ SANS data.

**Figure 10:**
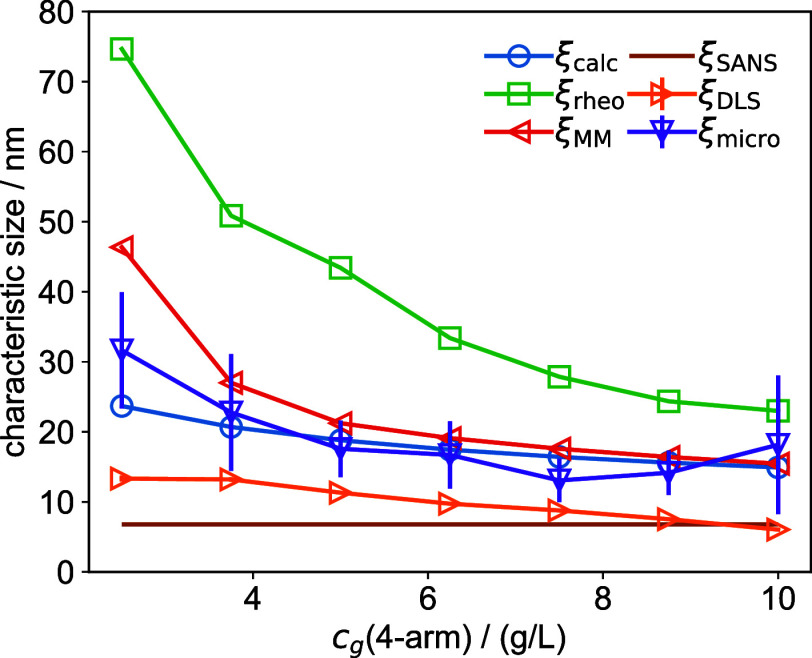
Characteristic sizes in soft PEG hydrogels, determined using various experimental techniques.

Depending on the cross-linker concentration and the method used, the characteristic sizes range between 6.76 and 74.7 nm. With the exception of ξ_SANS_, all characteristic sizes increase as the cross-linker concentration decreases.

ξ_SANS_ will be rather constant as it is just a measure of the local extension of the polymer chains connecting the different network cross-linking points and the same applies to ξ_DLS_, which measures the effective diffusion of these polymer blobs. The characteristic sizes can be grouped into three broad categories

- Scattering sizes (ξ_SANS_, ξ_DLS_): Reflect the correlation length observed in scattering experiments, i.e., the effective extension of the local polymer chains.
- Connectivity sizes (ξ_calc_, ξ_MM_, ξ_micro_): Relate to the network structure and cross-linking density.
- Rheology size (ξ_rheo_): Reflects the elastic properties of the hydrogel network.

These categories correspond to what Tsuji et al. referred to as the correlation blob, geometric blob, and elastic blob, respectively.^36^ We find that, in general, rheology size > connectivity sizes > correlation sizes, in agreement with previous findings.^36^ ξ_micro_ should reflect an experimental measure of the theoretical estimates for the mesh size, ξ_calc_ and ξ_MM_, as it measures the average distance a particle can move inside the hydrogel, before becoming trapped in the network. Although our tracer particles are much larger than all of the characteristic sizes in the gel, ξ_micro_ matches closely with the theoretically estimated connectivity sizes.

The differences between the characteristic length scales are most pronounced at low 4-arm concentrations but gradually converge toward similar values as the 4-arm concentration increases. This trend can be understood when looking at the idealized sketches in Figure 1B. At high 4-arm concentrations (e.g., T_1_), the average spacing between elastically effective cross-links (ξ_rheo_)­and the average spacing between scattering polymer chains (ξ_SANS_) become nearly equivalent. Similar reasoning applies to the other characteristic length scales. In this highly connected regime, the network structure is effectively homogeneous, with a single dominant characteristic size. As the 4-arm concentration decreases (e.g., T_0.25_), the spacing between scattering chains remains relatively constant, while the spacing between elastically effective cross-links increases. This divergence explains the growing spread among the various characteristic sizes at lower concentrations. The disparity between the correlation sizes (from scattering) and the other sizes highlights the influence of elastically ineffective polymers in the hydrogel, such as linear chains or loops, which contribute to the scattering but not to the rheological properties. Scattering-derived sizes, therefore, are not reliable measures of the hydrogel’s effective mesh size. Moreover, while the absolute values of ξ_rheo_ and ξ_MM_ differ, the Miller–Macosko method is the only one that successfully captures the steep increase seen in ξ_rheo_ as the cross-linker concentration approaches the gelation threshold.

## Conclusion

This paper focuses on the preparation, characterization and theoretical description of soft hydrogels, based on the tetra-PEG method, with a defined network structure. This study demonstrated the adaptation of tetra-PEG hydrogels to soft regimes by introducing linear precursors, achieving precise control over elastic moduli while maintaining network integrity. This approach broadens the applicability of tetra-PEG hydrogels by allowing to mimic the rheological and structural properties of biological hydrogels. As an example, in Figure 11, the frequency sweeps of sample T_0.25_ are compared to those of mucus from healthy individuals and patients with the muco-obstructive lung disease cystic fibrosis (CF).

**Figure 11:**
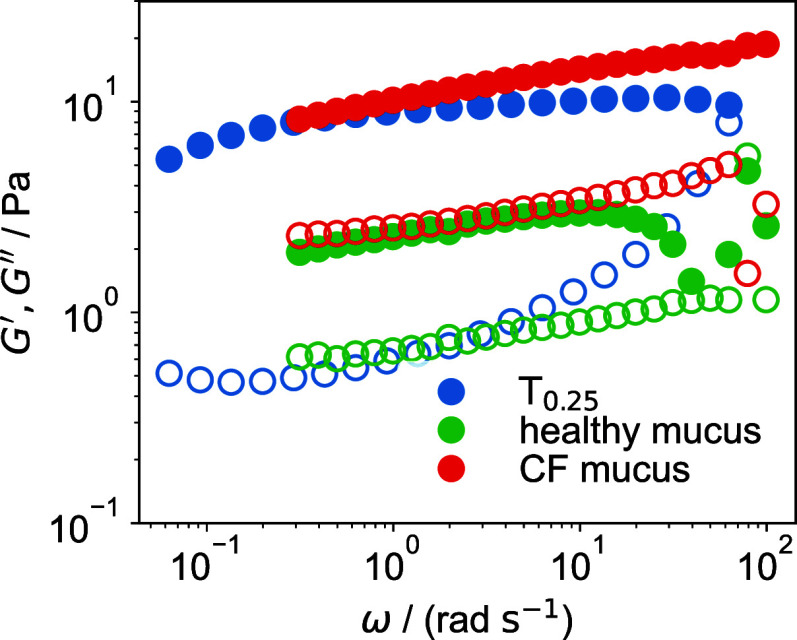
Frequency sweeps of T_0.25_ compared with healthy mucus and CF mucus. Full and broken symbols denote *G*′ and *G*″^′^, respectively. The data acquisition of the mucus samples is explained in detail in ref 28.

The data from our simple modified tetra-PEG hydrogels show rheological properties that are comparable in magnitude to those observed in mucus. The agreement is not exact, particularly in terms of loss tangent, $\tan\delta =G^{\prime \prime }/G^{\prime }$ , which appears significantly higher in mucus. This is expected given the considerably greater structural and biochemical complexity of native mucus, consisting of glycoproteins with specialized biological functions. Nevertheless, these modified tetra-PEG hydrogels may serve not only as reproducible model systems for probing the fundamental physics of mucus-like materials but they also offer insights for the rational design of advanced biomaterials for biomedical applications such as drug delivery, bioadhesion, and soft tissue engineering.

Using UV-spectroscopy, the maleimide–thiol reaction conversion could be followed. The conversion was found to be around 86% and rather independent of the sample composition. Rheological experiments confirmed the formation of stable elastic networks for samples with sufficient cross-linker content (T_1_–T_0.25_), where *G*′ ≫ *G*″. *G*′ was found to increase approximately linearly with the cross-linker content.

The Miller–Macosko approximation was able to account for the multicomponent system. The model correctly predicts the gelation threshold concentration of 2.5 g/L cross-linker. However, the large discrepancy in the absolute values between experiment and model (ratio of approximately 0.3) suggests that elastically ineffective structures such as loops appear frequently, likely due to the overall rather low polymer content of 10 g/L, below the overlap concentration.

The collective hydrogel dynamics were characterized using DLS. Due to the permanent chemical cross-links, the hydrogels show nonergodic behavior, which was explicitly accounted for. The hydrogels exhibited one dominant relaxation mode at approximately 10^–4^ s, associated with the elastic deformation of network strands.

By adding polystyrene tracer particles, DLS microrheology experiments were conducted. The particle MSD leveled off to a constant value for *t* → ∞, indicating that the particles were confined to a finite region, thereby yielding a measure of the effective mesh size. We showed that the DLS microrheology technique can hardly distinguish between stiff hydrogels, where *G*′ >100 Pa, since the corresponding correlation functions show only an extremely small decrease from 0.01 to 0, which cannot be precisely resolved in the experiment.

The SANS curves of the modified tetra-PEG samples are nearly identical for intermediate and high *q* values indicating that the local structure is very similar, irrespective of composition. The influence of large-scale inhomogeneities was found to increase approximately linearly with the cross-linker content.

Using this combination of experimental techniques, several characteristic sizes could be determined that can be grouped into three categories: correlation sizes (ξ_SANS_ and ξ_DLS_), connectivity sizes (ξ_calc_, ξ_MM_ and ξ_micro_) and the rheology size (ξ_rheo_). The correlation sizes are significantly smaller than the rest, because elastically ineffective features contribute to scattering but not to the rheological properties. Scattering-based techniques are therefore not an accurate representation of the mesh size of such hydrogels.

In summary, we demonstrate how the tetra-PEG framework can be adapted to produce hydrogels with very low and well-controlled rheological moduli. In this way one can obtain model networks that can be used to compare to the properties of more complex biological hydrogels, thereby yielding more systematic and deepened insights into their properties. This concept could potentially be extended in the future by including PEG precursor macromers with other geometries such as 3-arm or 5-arm star polymers. The Miller–Macosko approximation could be adapted to any combination of geometries in a similar ways as shown in this paper.

## Supplementary Material





## Data Availability

The experimental data are available from the authors upon reasonable request.

## Notes

Sample preparation; calculation of end-to-end distances; UV–vis details; rheology details (including amplitude sweeps and GMM fits); Miller–Macosko approximation adapted for modified tetra-PEG hydrogels (including possible loop structures); DLS details; microrheology details; SANS details (PDF)
Very soft hydrogel T_0.25_ (MP4)
