# Supplementary material for: Fabrication and Characterization of Tetra-PEG-Derived Hydrogels of Controlled Softness
Source: Macromolecules. 2025 Jun 25;58(13):6916–28. doi: 10.1021/acs.macromol.5c00695 (PMC12257590; doi:10.1021/acs.macromol.5c00695)
Supplement: Supplementary file 1 [file ma5c00695_si_001.pdf]

SUPPORTING INFORMATION for: Fabrication and Characterization of Ultra-Soft tetra-PEG-derived Hydrogels

Robert F. Schmidt,[*,†] Olga Matsarskaia,[‡] Takamasa Sakai,[¶] and Michael Gradzielski[†]

†*Stranski-Laboratorium für Physikalische und Theoretische Chemie, Strasse des 17. Juni 124, 10623 Berlin, Germany*

‡*Institut Laue-Langevin, 71 Avenue des Martyrs, CS 20156, 38042 Grenoble Cedex 9, France*

¶*Department of Chemistry & Biotechnology, School of Engineering, The University of Tokyo, Tokyo 113-8656, Japan*

E-mail: r.schmidt.1@tu-berlin.de

Sample Preparation

10 g/L stock solutions of the four precursor macromers 4-arm PEG-thiol (20 kDa, PTE-200SH, NOF Corporation, Japan, LOT M236681), 2-arm PEG-thiol (10 kDa, DE-100SH, NOF Corporation, Japan, LOT M138544), 4-arm PEG-maleimide (20 kDa, PTE-200MA, NOF Corporation, Japan, LOT M239622), 2-arm PEG-maleimide (10 kDa, DE-100MA, NOF Corporation, Japan, LOT M15N590) were prepared in citrate-phosphate buffer (CPB) of pH 3.4. The ionic strength and pH of the buffer were chosen to control the reaction rate of the thiol-maleimide Michael addition reaction.[1–3] Since the maleimide group degrades over time,

the stock solutions have to be used immediately and cannot be stored. For 100 mL buffer, 1.08 g citric acid and 0.78 g disodium hydrogen phosphate dihydrate ($Na_2HPO_4x2\,H_2O$) were dissolved in 100 mL Milli-Q water to obtain the desired pH 3.4 and a buffer capacity of 0.1 M. The stock solutions were mixed in appropriate ratios to obtain the desired final composition. An overview over all samples and their compositions is given in Table 1. After vortexing for 30 s, the samples were left to react at room temperature for at least 24 h. Depending on the experiment, the general procedure is slightly changed. For rheology and microrheology experiments polystyrene particles (Polybead Microspheres, 0.2 µm, 2.5% solids (w/v), Polysciences) with a diameter of 192 nm were added to the buffer to obtain a final particle concentration of 0.003 %w/v. Directly after vortexing, each sample was divided into two parts, one for microrheology and one for macrorheology. The microrheology part was filled into filled into 17.78 cm long glass cuvettes with a diameter of 1 cm. The macrorheology part was filled into a cylindrical plastic jar. The DLS samples (without particles) were also filled into the long glass cuvettes. The SANS samples were prepared in an analogous fashion but with D_2O instead of H_2O to allow for better contrast. The D_2O was filtered using a 20 µm cellulose acetate filter prior to use. No PS particles were added for the SANS experiments.

Table 1: Overview of samples and their compositions. All mass concentration values, c_g, are given in g/L.

sample	c_g(4SH)	c_g(2SH)	c_g(4Mal)	c_g(2Mal)	$\sum c_g$	c_g(crosslinker)
T_1	5	0	5	0	10	10
$T_{0.875}$	5	0	3.75	1.25	10	8.75
$T_{0.75}$	5	0	2.5	2.5	10	7.5
$T_{0.625}$	5	0	1.25	3.75	10	6.25
$T_{0.5}$	5	0	0	5	10	5
$T_{0.375}$	3.75	1.25	0	5	10	3.75
$T_{0.25}$	2.5	2.5	0	5	10	2.5
$T_{0.125}$	1.25	3.75	0	5	10	1.25
T_0	0	5	0	5	10	0

Swelling Experiments

Determining swelling ratios for extremely soft hydrogels, such as those investigated in this study, is inherently challenging, as previously noted by Matsunaga *et al.*[4] To nonetheless obtain a rough estimate of the equilibrium swelling ratio, we employed the strategy illustrated in Figure1A. Four gels were tested, namely T_1, $T_{0.75}$, $T_{0.5}$ and $T_{0.25}$.

A piece of gel in its initial, as-prepared state was placed inside a cell strainer (Corning, pore size 100 µm), and its initial mass, m_i, was measured using a scale. Given a polymer concentration of $c_g = 10\,g/L$ in the initial state, the dry polymer mass is estimated as $m_d \approx 0.01 m_i$, assuming the density of the gel matches that of water, $\rho_{gel} = \rho_{water} = 1\,g/mL$.

The gel-containing strainer was then submerged in 30 mL of CPB buffer, and the container was sealed with Parafilm. The sample was allowed to swell for 3 days in a dark cupboard at ambient temperature. After this period, the strainer was removed, placed on a piece of tissue paper, and left to drain and dry for 45 minutes. The mass of the swollen gel, m_s, was then recorded.

From these measurements, two swelling ratios were calculated: the ratio of swollen to initial mass, $Q_i = m_s/m_i$, and the ratio of swollen to dry mass, $Q_d = m_s/m_d$. The resulting swelling ratios are presented in Figure 1B.

We observed that the stiffest hydrogel, T_1, exhibited a decrease in mass from the initial to the swollen state. This outcome is highly unexpected and likely reflects artifacts in the swelling procedure rather than a true physical reduction in gel mass. A genuine decrease would only be plausible if the gel had a very large sol fraction (unincorporated or weakly connected polymer fragments) that was leached out during swelling. However, more probable explanations include the accidental loss of gel fragments during handling or leakage of small pieces through the pores of the cell strainer.

Despite this anomaly, the mass swelling ratio, Q_i, shows a consistent increasing trend as the polymer concentration c_g(4-arm) decreases, reaching a value of 2.24 for $T_{0.25}$. This trend aligns with expectations, as a looser network structure with larger mesh sizes enables greater

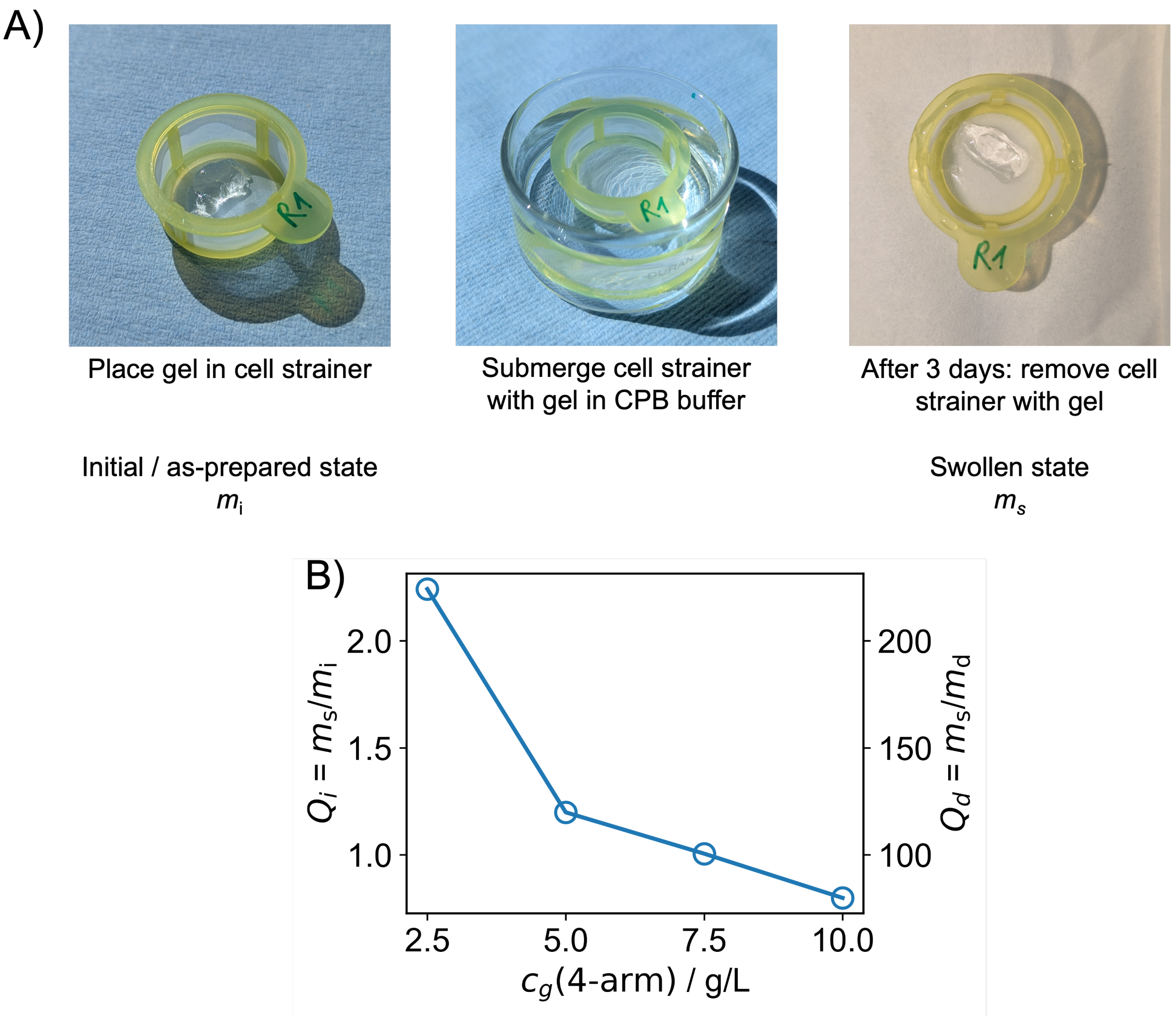

Figure 1: A) Procedure for swelling experiments. A piece of gel in the initial, as-prepared state (mass of gel piece: m_i) is placed inside a cell strainer and then submerged in the CPB buffer for 3 days. Afterwards, the mass of the gel in the swollen state, m_s, is determined. B) Swelling ratios Q_i and Q_d.

solvent uptake and, consequently, higher swelling ratios. The swelling ratio with respect to the dry mass reaches values between 80 and 224 for T_1 and $T_{0.25}$, respectively.

UV-Vis

The UV-Vis experiments were performed on a V-670 Spectrophotometer (Jasco, Tokyo, Japan). The absorption spectra of the pure precursor solutions were measured between 230 nm and 600 nm wavelength. Four individual scans with measurement times of 60 s were collected and averaged. For the time evolution experiments, the appropriate masses of 4-arm PEG-thiol (Sinopeg, China, Item No. 06020701312, LOT P1911070131203-B), 2-arm PEG-thiol (NOF Corporation, Japan, Item DE-100SH, LOT M138544), 4-arm PEG-maleimide (NOF Corporation, Japan, Item No. PTE-200MA, LOT M239622) and 2-arm PEG-maleimide (NOF Corporation, Japan, Item No. DE-100MA, LOT M15N590) to prepare 5 mL of a T_x sample were put into a cylindrical plastic jar. Right before starting the measurement, the 5 mL of CPB buffer were added and the sample was vortexed for 2 min. 1 mL of the sample was then filled into a 1.5 mL disposable plastic cuvette with a path length of 1 cm and placed into the spectrophotometer. The total time between mixing and starting the measurement was 4 min. The absorption at a fixed wavelength of 300 nm was then measured for 24 h with one data point being collected after each minute. The absorbance of the solvent and the cuvettes were subtracted. The measured absorbances A were converted into the extinction using Lambert–Beer's law:

$$\epsilon = \frac{A}{c_m \cdot l}\,, \tag{1}$$

where c_m is the molar concentration (corresponding here to the number of SH or Mal moieties, not the number of precursor macromers) and l is the path length, which was 1 cm in all cases.

For the conversion calculation shown in Figure 3C in the main text, the absorbance of the maleimide species at time t is needed. At 300 nm, the thiol species still absorb a little

bit of light, as seen in Figure 3B. To correct for this, the absorbance of the thiol species is subtracted according to:

$$A_{\mathrm{Mal,300nm}}(t) = A_{\mathrm{300nm}}(t) - \frac{c_g(\mathrm{2SH{+}4SH})}{10\,\mathrm{g/L}} A_{\mathrm{SH,300nm}}\,. \tag{2}$$

Eq. 2 assumes that the absorbance of the thiol molecules is not affected by their reaction with the maleimide species.

Rheology

Rheological measurements were performed on an MCR 502 WESP temperature-controlled rheometer from Anton Paar (Graz, Austria) in strain-imposed mode. A parallel plate measuring system with a diameter of 25 mm was used. The gels are too soft to be prepared inside a mold and pipetting them would create strong shear forces that could destroy the delicate network. Therefore, the gels were transferred onto the lower rheometer plate using a spatula leading to variable amounts of gel in the rheometer. The gap width was adjusted for each sample to ensure that the gap was properly filled and that the gel was in contact with the plate. It varied between 1 mm and 0.3 mm. Amplitude sweeps were performed at a constant frequency of 6.28 rad/s for strain amplitudes between 0.1 and 10%. The are shown below in Figure 2. Frequency sweeps were performed at a constant strain amplitude of 5% and for frequencies between 0.063 and 63 rad/s, first in order of increasing frequencies (up-sweep), then in order of decreasing frequencies (down-sweep). Since there were no significant differences between the two frequency sweeps, only the up-sweep is considered in the main text.

Amplitude Sweeps

Amplitude sweeps were performed at a constant frequency of 6.28 rad/s. The results are shown in Figure 2. Since both moduli are constant over the tested strain amplitude range,

we are in the LVE range. All frequency sweep measurements were performed with a strain amplitude of $\gamma_0 = 5\,\%$, as indicated by the dashed black line in Figure 2.

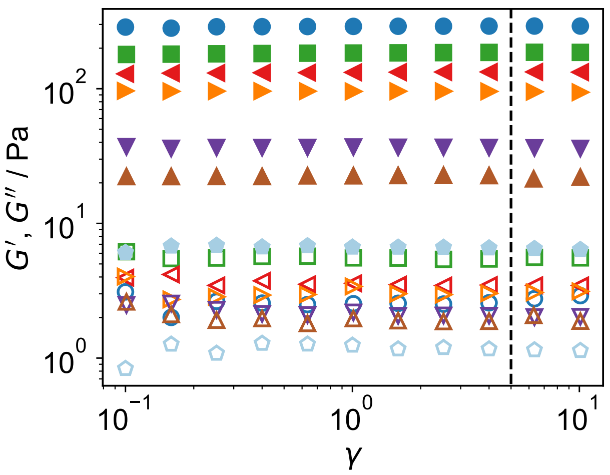

Figure 2: Amplitude sweeps of the modified tetraPEG samples. All frequency sweeps, performed at $\gamma_0 = 5\,\%$, are in the LVE range.

Loss Tangents

The loss tangents of the frequency sweep measurements, $\tan\delta = G''/G'$, are shown in Figure 3. The loss tangents are all smaller than 1, indicating that the elastic properties dominate.

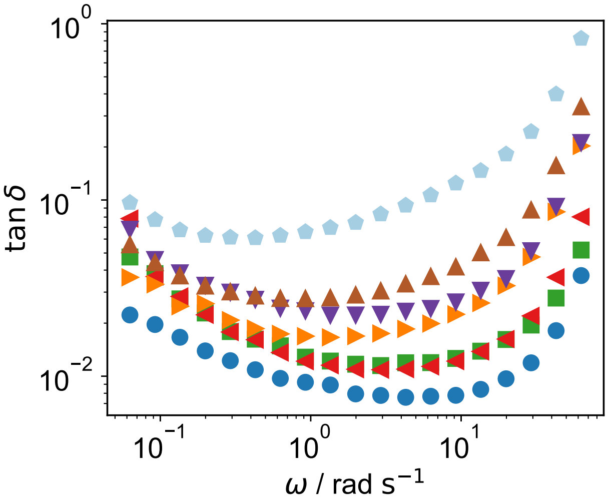

Figure 3: Loss tangents of the tetra-PEG gel samples.

$\tan\delta$ is lowest for the stiffest hydrogel, T_1 and increases with decreasing cross-linker concen-

tration. As shown in the main text, G'' remains rather low, irrespective of the composition, meaning the variation in $\tan\delta$ is mostly explained by the variation in G'.

Miller-Macosko Approximation Adapted for Modified Tetra-PEG Hydrogels

The tree-like approximation for a system consisting of two 4-arm and two 2-arm species is depicted in the main text in Figure 5 and shown again here in Figure 4 for convenience.

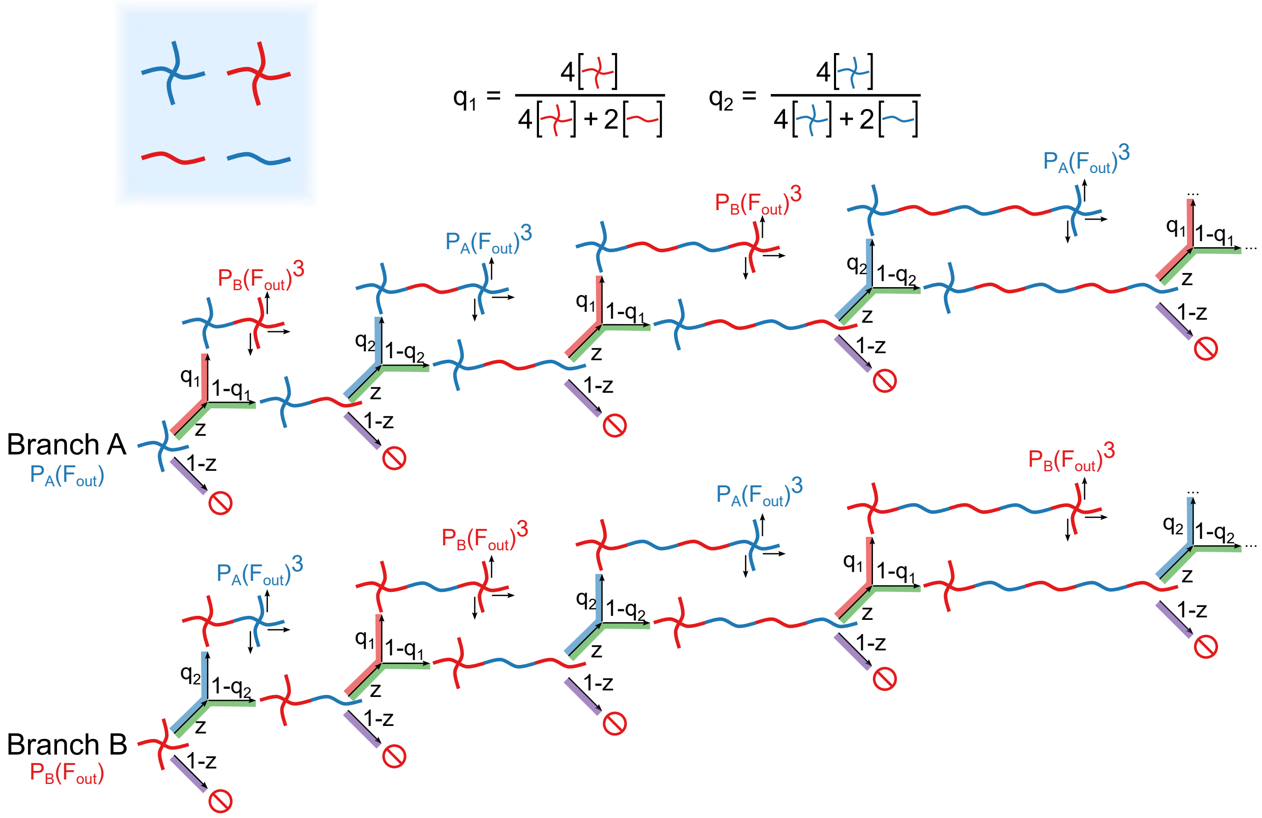

Figure 4: Visualization of the Miller-Macosko theory for a system consisting of two 4-arm molecules and two 2-arm molecules. There is an infinite number of possible paths, which can be divided into three groups. The stop paths are those which lead to a dangling chain (highlighted in purple). The linear path corresponds to the formation of an infinitely long linear chain (highlighted in green). The recursive paths lead back to A or B (highlighted in blue or red, respectively).

We define the probability that a thiol molecule (4SH or 2SH) reacts with 4Mal as $q_1 =$

$\frac{4c_m(\text{4Mal})}{4c_m(\text{4Mal}+2c_m(\text{2Mal}}$, where [...]. The probability to react with 2Mal then is $1-q_1$. Conversely, the probabilities that a maleimide molecule (4Mal or 2Mal) reacts with 4SH and 2SH are $q_2 = \frac{4c_m(\text{4SH})}{4c_m(\text{4SH})+2c_m(\text{2SH})}$ and $1-q_2$, respectively. We therefore make the following assumptions: the probability for any reaction to occur is always equal to z. q_1 and q_2 are constant throughout the reaction process, meaning we do not account for the case that one of the two species is consumed faster than the other. As seen in Figure 4, there is an infinite number of possible paths. We proceed by dividing the paths into three groups, which are highlighted with different colors. The stop-paths are those which lead to a dangling chain (purple). The linear path corresponds to the formation of an infinitely long linear chain (green). The recursive paths lead back to A or B (blue or red, respectively). The probability for the stop paths can be written as

$$\begin{aligned}
P_{\text{stop}} &= (1-z)\left[1 + z(1-q_1) + z^2(1-q_1)(1-q_2) + z^3(1-q_1)^2(1-q_2) + \dots\right] \\
&= (1-z)\left[1 + \left(z(1-q_1) + z^2(1-q_1)(1-q_2)\right)\sum_{k=0}^{\infty}[z^2(1-q_1)(1-q_2)]^k\right] \\
&= \frac{(1-z)(1+z-zq_1)}{1-z^2(1-q_1)(1-q_2)} \\
&= C_1\,,
\end{aligned} \tag{3}$$

where we have used that the infinite series is a geometric series. Similarly, the recursive paths leading back to A (blue paths) and B (red paths) can be summarized as

$$\begin{aligned}
P_{\text{recursion,A}} &= z^2(1-q_1)q_2 P_A(F_{\text{out}})^3\left[1 + \left(z^2(1-q_1)(1-q_2)\right)\sum_{k=0}^{\infty}[z^2(1-q_1)(1-q_2)]^k\right] \\
&= \frac{z^2(1-q_1)q_2}{1-z^2(1-q_1)(1-q_2)} P_A(F_{\text{out}})^3 \\
&= C_2 \cdot P_A(F_{\text{out}})^3
\end{aligned} \tag{4}$$

and

$$
\begin{aligned}
P_{\text{recursion,B}} &= zq_1 P_B(F_{\text{out}})^3 \left[1 + \left(z^2(1-q_1)(1-q_2)\right) \sum_{k=0}^{\infty} [z^2(1-q_1)(1-q_2)]^k\right] \\
&= \frac{zq_1}{1 - z^2(1-q_1)(1-q_2)} P_B(F_{\text{out}})^3 \\
&= C_3 \cdot P_B(F_{\text{out}})^3 \,,
\end{aligned} \tag{5}
$$

respectively. The probability for the linear path is given by

$$
P_{\text{linear}} = \prod_{i=1}^{\infty} z^2(1-q_1)(1-q_2)\,, \tag{6}
$$

which goes to zero for a macroscopic system. Combining equations 3, 4 and 5 yields

$$
P_A(F_{\text{out}}) = C_1 + C_2 \cdot P_A(F_{\text{out}})^3 + C_3 \cdot P_B(F_{\text{out}})^3 \,. \tag{7}
$$

The probabilities for Path B can be determined in an analogous fashion leading to

$$
P_B(F_{\text{out}}) = C_4 + C_5 \cdot P_B(F_{\text{out}})^3 + C_6 \cdot P_A(F_{\text{out}})^3 \,, \tag{8}
$$

where C_4, C_5 and C_6 are defined in the same way as C_1, C_2 and C_3, respectively, but with interchanged q_1 and q_2. Equations 7 and 8 constitute a set of coupled nonlinear equations, which can be solved numerically.

Prediction of Sol Content and Fraction of Dangling Chain Ends

To other important parameters, which can be determined from the Miller-Macosko theory are the sol content, w_{sol}, and the number fraction of connections which lead to dangling ends, x_{dangling}. The sol content refers to the mass fraction of the polymeric material, which is not connected to the infinite network and will therefore be washed out if the gel is placed in a

solvent. For our system, the sol fraction is given by:[5,6]

$$w_{\text{sol}} = w_{\text{4SH}} P_A(F_{\text{out}})^4 + w_{\text{4Mal}} P_B(F_{\text{out}})^4 + w_{\text{2SH}} P_C(F_{\text{out}})^2 + w_{\text{2Mal}} P_D(F_{\text{out}})^2\,, \tag{9}$$

where w_i denotes the weight fraction of precursor macromer i in the mixture. $P_C(F_{\text{out}})$ and $P_D(F_{\text{out}})$ are the probabilities that starting at a 2SH and a 2Mal molecule and following one arm leads to a finite chain, respectively. The corresponding branches C and D are shown in Figure 5.

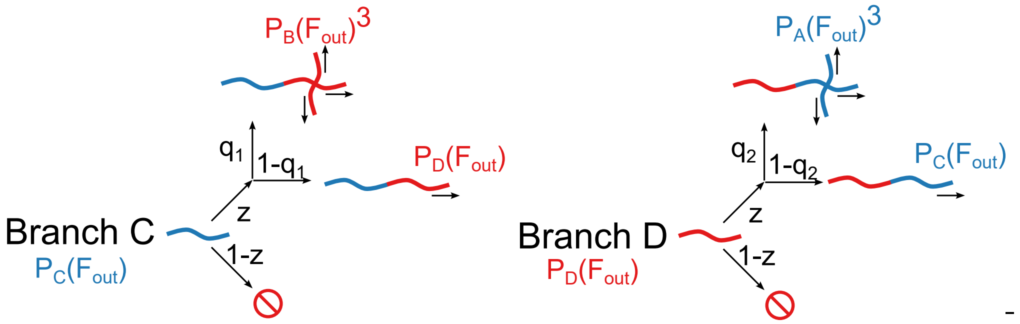

Figure 5: Branches C and D, describing the probability that following an arm of 2SH or 2Mal lead to a finite chain, respectively.

Adding up the probabilities we find

$$P_C(F_{\text{out}}) = 1 - z + zq_1 P_B(F_{\text{out}})^3 + z(1-q_1)P_D(F_{\text{out}}) \tag{10}$$

and

$$P_D(F_{\text{out}}) = 1 - z + zq_2 P_A(F_{\text{out}})^3 + z(1-q_2)P_C(F_{\text{out}})\,. \tag{11}$$

After $P_A(F_{\text{out}})$ and $P_B(F_{\text{out}})$ have been determined numerically according to the procedure outlined above, $P_C(F_{\text{out}})$ and $P_D(F_{\text{out}})$ can be calculated analytically by combining Eqs. 10 and 11.

x_{dangling} can be estimated via

$$x_{\text{dangling}} = \frac{4c_{\text{m,4SH}} P_A(F_{\text{out}}) + 4c_{\text{m,4Mal}} P_B(F_{\text{out}}) + 2c_{\text{m,2SH}} P_C(F_{\text{out}}) + 2c_{\text{m,2Mal}} P_D(F_{\text{out}})}{4c_{\text{m,4SH}} + 4c_{\text{m,4Mal}} + 2c_{\text{m,2SH}} + 2c_{\text{m,2SH}}}\,, \tag{12}$$

$c_{\mathrm{m,i}}$ denoting the molar concentration of precursor macromer i. w_{sol} and x_{dangling} are shown in Figure 6. Below a threshold of around 1.0 g/L, $w_{\mathrm{sol}} = x_{\mathrm{dangling}} = 1$, indicating the complete breakdown of the network. For c_g(4-arm) = 2.5 g/L, w_{sol} decreases to 0.15, meaning even for the softest measurable gel, only around 15% of the polymeric material is not connected to the infinite network. Beyond c_g(4-arm) = 5 g/L, the sol fraction becomes very small and approaches 0. x_{dangling} decreases to around 0.43 for c_g(4-arm) = 2.5 g/L, meaning almost half of the connections lead to dangling chain ends. Beyond c_g(4-arm) = 5 g/L, x_{dangling} is at around 0.2 and decreases only very slowly, indicating that even in the stiffest gels, every fifth connection leads to a dangling chain end.

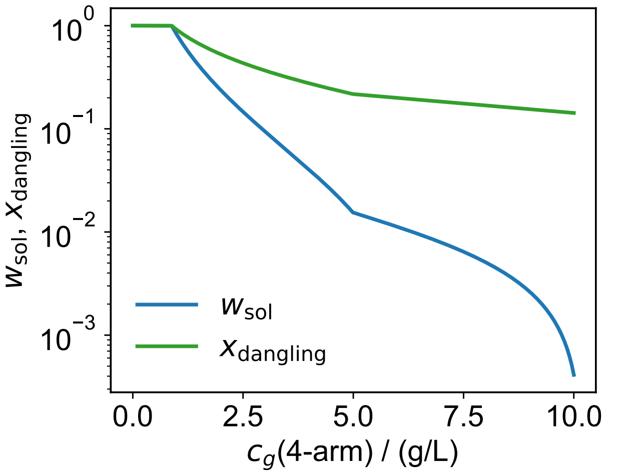

Figure 6: Predicted mass fraction of soluble content, w_{sol}, and number fraction of dangling chain ends, x_{dangling}, as a function of the concentration of 4-armed molecules.

Possible Closed Loop Structures

The deviation between the predicted and the experimentally measured moduli suggest that a significant amount of elastically ineffective structures are formed, which include closed loops. Some examples of possible closed loop structures are shown in Figure 7.

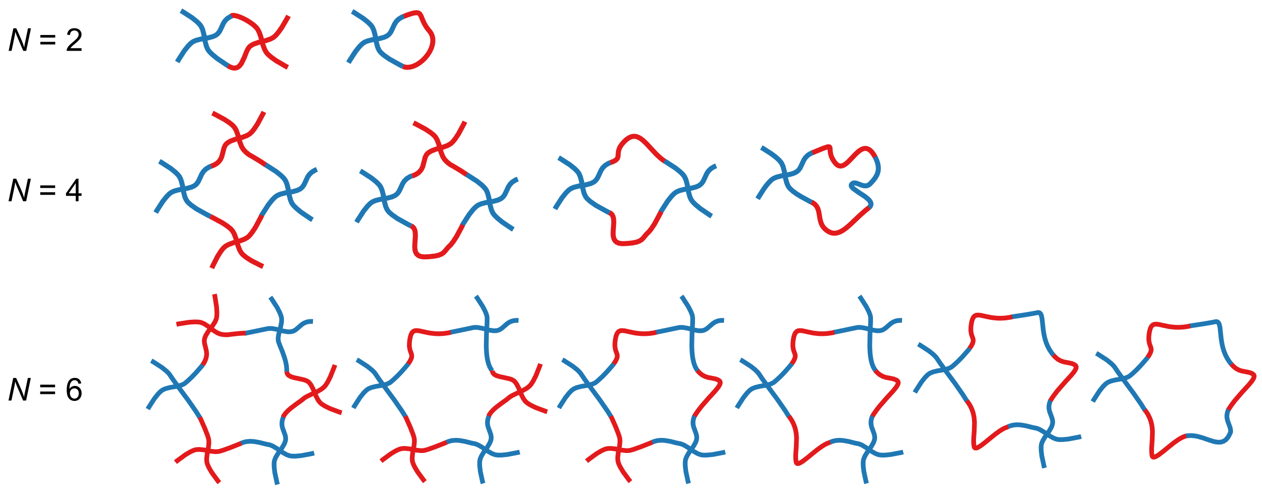

Figure 7: Possible closed loop configurations for $N = 2$, 4 or 6 molecules.

Closed loops can only be formed for even numbers of molecules because the thiol and maleimide groups cannot react with themselves. The probability for the formation of N-membered rings becomes increasingly small as N increases, because the N-th molecule will be, on average, quite far away from the original molecule, making a reaction unlikely. A ring by itself can be considered elastically effective if three or more of the outreaching arms are connected to the infinite network. Within the Miller-Macosko framework, there is no way of explicitly accounting for the formation of loops. Other theoretical approaches, such as the spanning-tree model, account for loops, but they are developed for homopolymerizations and adapting them to our multi-component system would be very tedious.[7?]

Dynamic Light Scattering

Light scattering experiments were performed on an ALV CGS-3 goniometry system (ALV, Langen, Germany), equipped with a He-Ne laser ($\lambda = 632.8$ nm at 22 mW) and a rotation/translation unit (CRTU). The correlation functions were recorded using an ALV 5000/E multiple-τ correlator at scattering angles of 30°, 50°, 70°, 90°, 110°, 130°. At each angle, 50 measurements of 5 s duration were performed. Between each measurement, the cuvette was rotated for 3 s at a rotation speed of 0.5 rpm. The ensemble-averaged scattering intensity,

$\langle I(q)\rangle_E$, was determined as the average of the intensities of the 50 individual measurements. The coherence factor of our light scattering setup, $\bar{\beta}_c$, was determined from the measurement of the sol samples, $T_{0.125}$ and T_0, which are ergodic. The raw intensity autocorrelation functions, $g^{(2)}_{\text{raw}}(\Delta t) - 1$, were fitted with a stretched exponential function

$$g^{(2)}(\Delta t) = \beta_c \exp\left(-a_s(\Delta t)^{b_s}\right) \,, \tag{13}$$

for $\Delta t < 3 \cdot 10^{-5}$ s. Here, a_s and b_s are constants. The determined β_c values were averaged, yielding $\bar{\beta}_c = 0.278$.

Using $\bar{\beta}_c$, the raw intensity correlation functions were rescaled to yield $g^{(2)}(\Delta t) - 1 = (g^{(2)}_{\text{raw}}(\Delta t) - 1)/\bar{\beta}_c$. This way, $g^{(2)}(\Delta t) - 1$ should decay from 1 to 0 for an ergodic sample. All measurements, for which $g^{(2)}(0) - 1 > 1$ were declared as outliers. Moreover, to identify other outliers, the means and standard deviations of the remaining curves were calculated. Any curves, for which more than half of the data points lie outside the standard deviation were also declared as outliers. All $g^{(2)}(\Delta t) - 1$ curves are shown in Figure 8.

For the remaining measurements, the ensemble-averaged field correlation functions, $g^{(1)}(\Delta t)$ were then determined from the corresponding time-averaged intensity correlation function, $g^{(2)}(\Delta)$ using the non-ergodic approach, described in the main text. The $g^{(1)}(\Delta t)$ curves were then averaged and are shown in Figure 9.

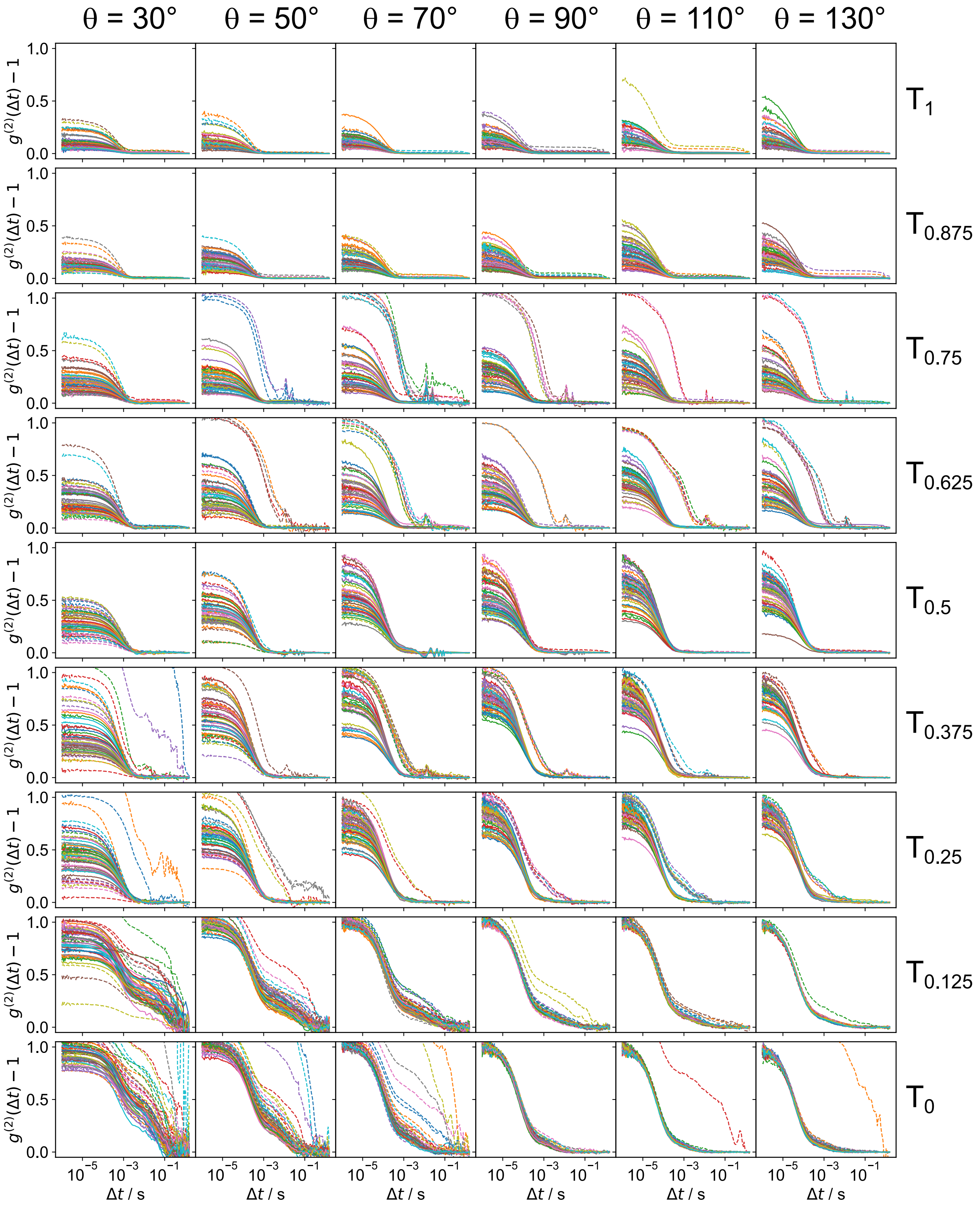

Figure 8: Rescaled intensity autocorrelation functions, $g^{(2)}(\Delta t) - 1 = (g^{(2)}_{\mathrm{raw}}(\Delta t) - 1)/\bar{\beta}_c$. Broken lines indicate measurements that were classified as outliers.

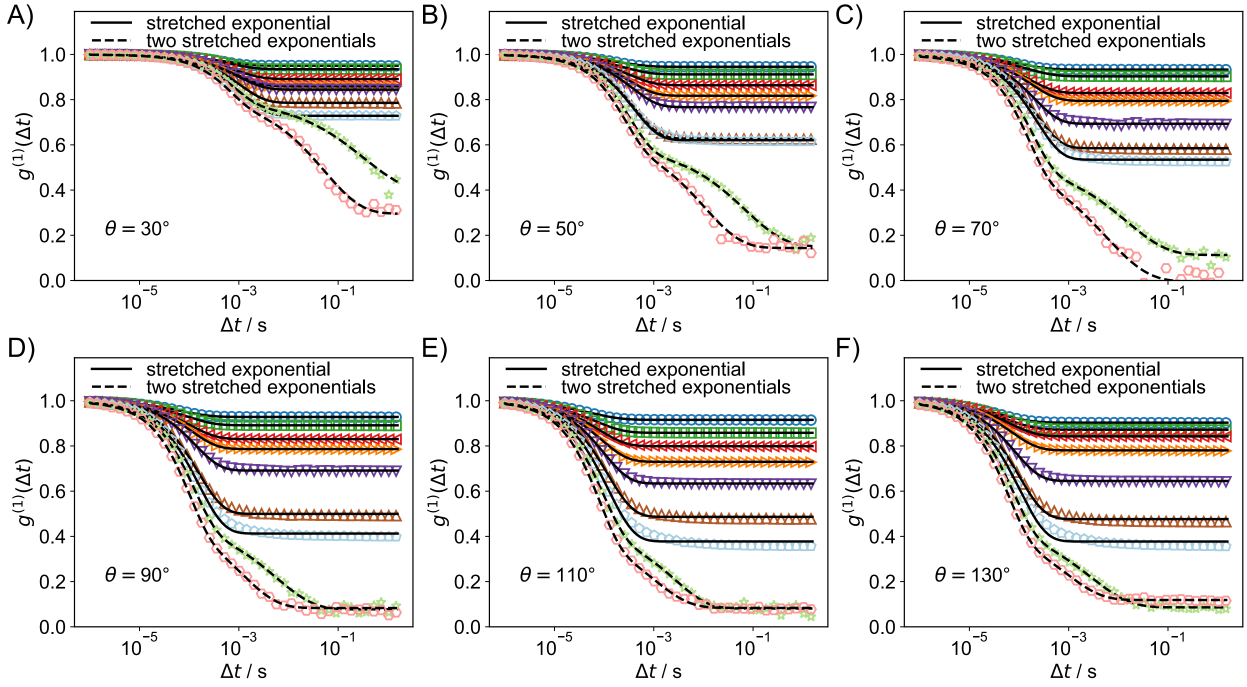

Figure 9: Field autocorrelation functions determined using the non-ergodic approach. The full and broken black lines indicate a fit with the Kohlrausch-Williams-Watts model with one (full lines) or two (broken lines) stretched exponentials.

As seen in Figure 9, the field autocorrelation functions for the two sol samples do not fully decay zero but to a small plateau value. This stems from deviations of the intercept values of $g^{(2)}(\tau)_{\mathrm{raw}} - 1$ from $be\bar{t}a_c$. If $g^{(2)}_{\mathrm{raw}}(0) - 1 < \beta_c$, then $g^{(2)}(0) - 1 < 1$. Eq. (14) in the main text for $\Delta t \to \infty$ is given by:

$$g^{(1)}(\Delta t \to \infty) = 1 + \frac{1}{Y}\left[\sqrt{g^{(2)}(\Delta t \to \infty) - g^{(2)}(0) - 1} - 1\right] \tag{14}$$

For a perfectly ergodic sample, the term in square brackets should approach -1, implying that $g^{(1)}(\Delta t \to \infty)$ converges to 0 when averaged over many positions ($Y = 1$ on average). However, for certain positions where $g^{(2)}_{\mathrm{raw}}(0) - 1 < \beta_c$, the term in square brackets exceeds -1. As a result, $g^{(1)}(\Delta)$ does not fully decay to zero. This behavior is likely due to inaccuracies in the DLS measurement and does not imply that these samples are non-ergodic.

Microrheology

Microrheology experiments were performed on the same ALV system used for DLS. The scattering angle was kept constant at $\theta = 90°$. To determine $\langle I(q)\rangle_E$, 100 measurements of 10 s each were performed while rotating the sample between each measurement for 2 s at 0.5 rpm. $g^{(2)}_{\text{raw}}(\Delta t)$ were collected from 10 measurements of 300 s duration, while rotation the sample for 12 s between each measurement. The raw correlation functions were rescaled using $\bar{\beta}_c(\theta = 90°)$. $g^{(1)}(\Delta t)$ was determined using non-ergodic approach. From $g^{(1)}(\Delta t)$, the MSD is calculated using the relation

$$g^{(1)}(t) = \exp\left(\frac{-q^2\langle\Delta r^2(t)\rangle}{6}\right) . \tag{15}$$

The MSD should always start at zero for $t = 0$. For some measurements, the obtained MSD has some residual value for very short times. To remove this residual value, the y-intercept of the MSD was determined from a linear fit for $10^{-6}\,\text{s} < t < 5 \cdot 10^{-6}\,\text{s}$ and subtracted from the MSD data. G' and G'' were then calculated from the MSD using the generalized Stokes-Einstein equation:[8,9]

$$G^*(\omega) = \frac{k_B T}{\pi a (i\omega)\langle\Delta\hat{r}^2(\omega)\rangle} , \tag{16}$$

where k_B is the Boltzmann constant, T is the absolute temperature, a is the hydrodynamic radius of the tracer particle and ω is the angular frequency. $\langle\Delta\hat{r}^2(\omega)\rangle$ denotes the Fourier transform of the MSD. We adopt the method of Mason *et al.*, whereby the MSD is expanded as a local power law according to $\langle\Delta r^2(t)\rangle \approx \langle\Delta r^2(1/\omega)\rangle(\omega t)^{\alpha(\omega)}$.[10] In that case, the Fourier transform can be calculated algebraically and the viscoelastic moduli are given by

$$\begin{aligned} G'(\omega) &= |G^*(\omega)| \cos\left[\pi\alpha(\omega)/2\right] \\ G''(\omega) &= |G^*(\omega)| \sin\left[\pi\alpha(\omega)/2\right] , \end{aligned} \tag{17}$$

with

$$|G^*(\omega)| = \frac{k_B T}{\pi a \langle \Delta r^2(1/\omega)\rangle \Gamma[1+\alpha(\omega)]}\,, \tag{18}$$

where $\Gamma(z)$ denotes the Gamma function. The gradient is determined numerically in Python using the `np.gradient()` function, which seeks to minimize the error between the true gradient and its estimate from a linear combination of neighboring points.[11–13]

The field correlation functions, MSDs and microrheological viscoelastic moduli, determined using the non-ergodic approach, are shown for all samples in Figures 10–18. For $T_{0.5}$, shown in Figure 14, oscillations are seen in $g^{(1)}(\tau)$, which propagate into the MSD and also G', G''. While less pronounced, signs of these oscillations can also be seen for $T_{0.625}$. The oscillation frequency of roughly 50 Hz is equal to the alternating current frequency in Germany, suggesting that the oscillations in the data are caused by electrical equipment in the vicinity of the light scattering instrument. It is unclear, why they appear only for some samples.

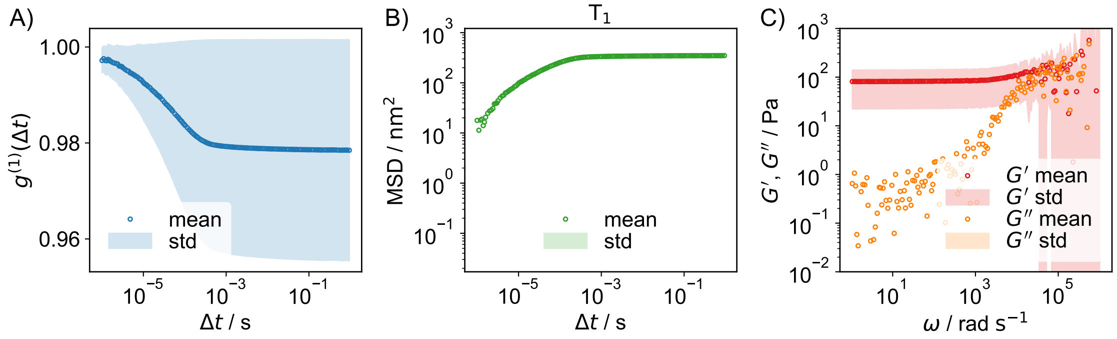

Figure 10: A) Field-field autocorrelation function, B) MSD and C) storage and loss moduli for T_1, determined using the non-ergodic approach.

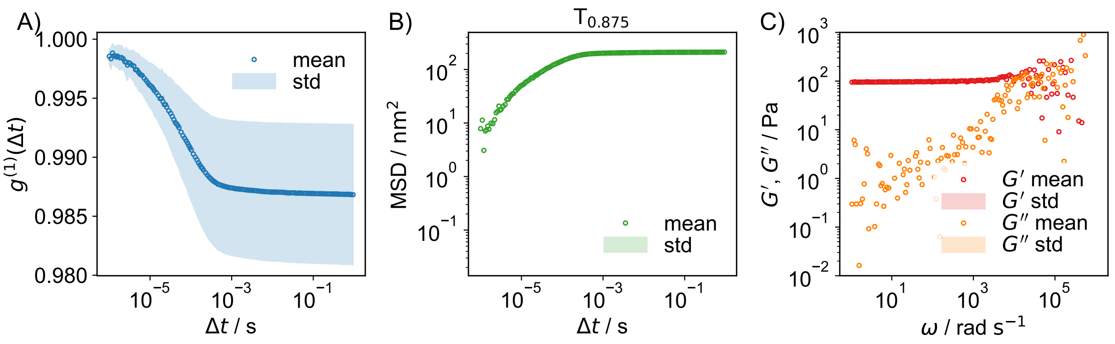

Figure 11: A) Field-field autocorrelation function, B) MSD and C) storage and loss moduli for $T_{0.875}$, determined using the non-ergodic approach.

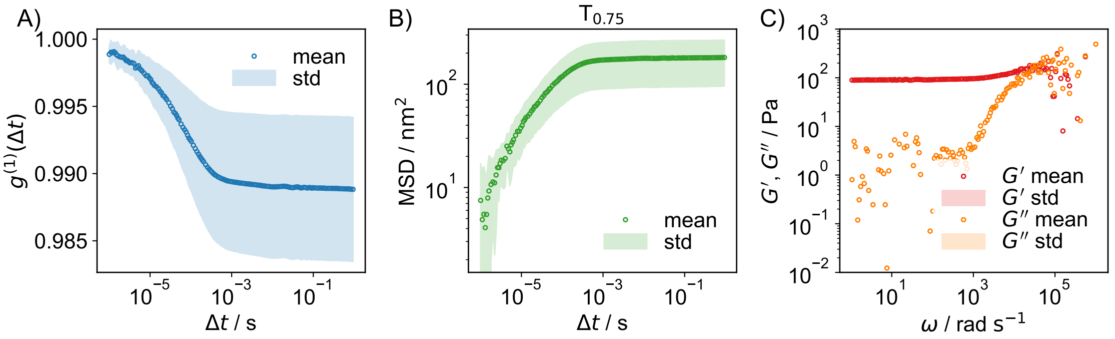

Figure 12: A) Field-field autocorrelation function, B) MSD and C) storage and loss moduli for $T_{0.75}$, determined using the non-ergodic approach.

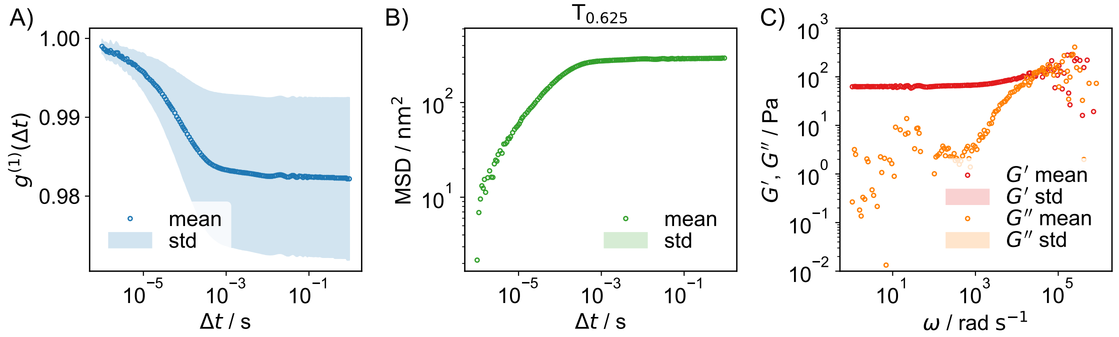

Figure 13: A) Field-field autocorrelation function, B) MSD and C) storage and loss moduli for $T_{0.625}$, determined using the non-ergodic approach.

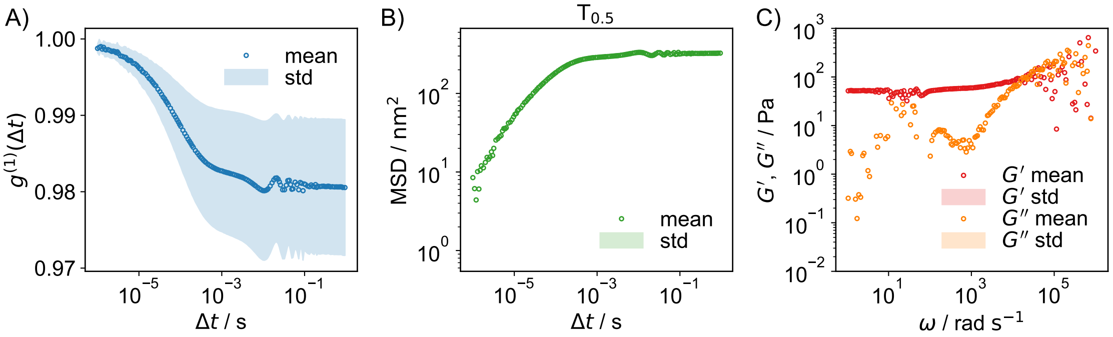

Figure 14: A) Field-field autocorrelation function, B) MSD and C) storage and loss moduli for $T_{0.5}$, determined using the non-ergodic approach.

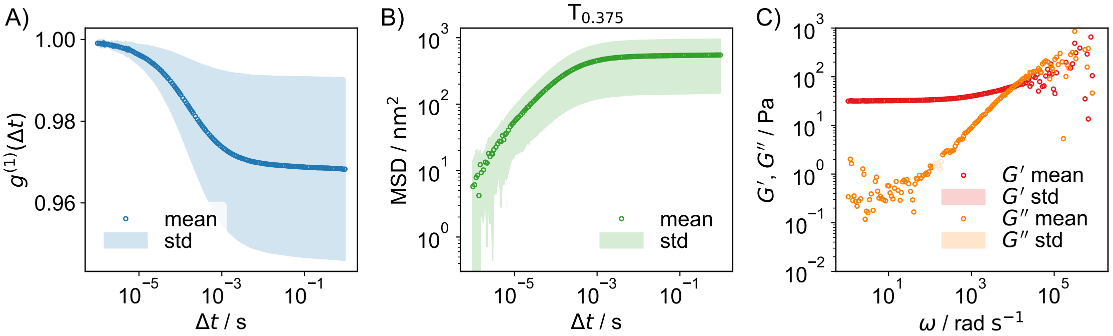

Figure 15: A) Field-field autocorrelation function, B) MSD and C) storage and loss moduli for $T_{0.375}$, determined using the non-ergodic approach.

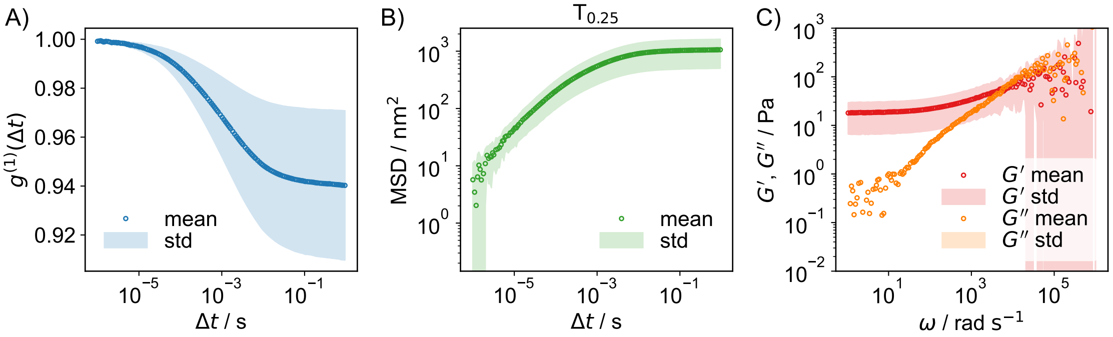

Figure 16: A) Field-field autocorrelation function, B) MSD and C) storage and loss moduli for $T_{0.25}$, determined using the non-ergodic approach.

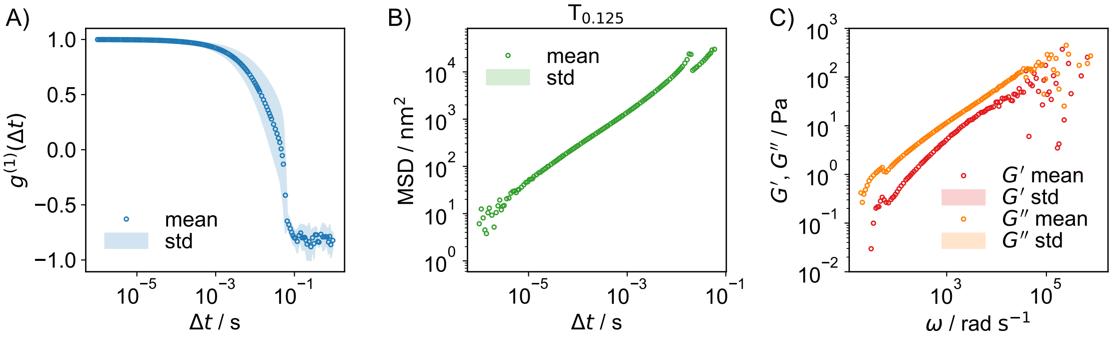

Figure 17: A) Field-field autocorrelation function, B) MSD and C) storage and loss moduli for $T_{0.125}$, determined using the non-ergodic approach.

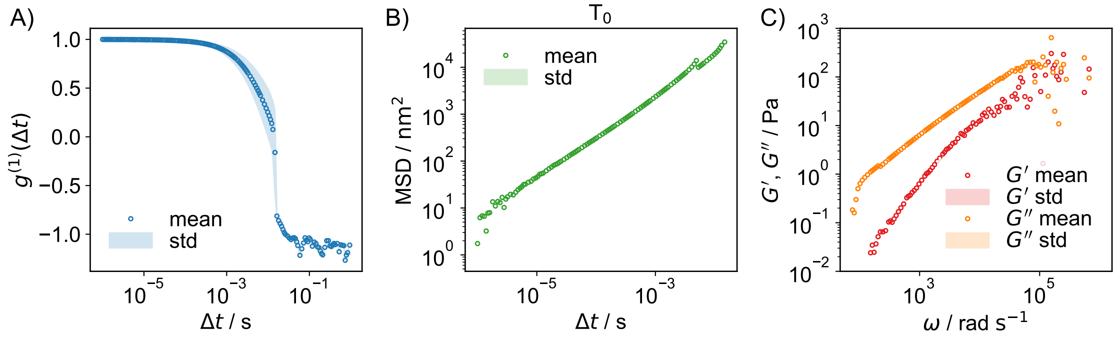

Figure 18: A) Field-field autocorrelation function, B) MSD and C) storage and loss moduli for T_0, determined using the non-ergodic approach.

Small-Angle Neutron Scattering

The SANS experiments were performed on the D33 instrument at ILL (experiment 9-10-1840[14]). The tetra-PEG samples were placed inside special sandwich cells, shown in Figure 19. Two round quartz plates are placed inside two brass holders with matching round cutouts. A FKM (fluoroelastomer) ring with a thickness of 1.2 mm is placed between the quartz plates and the gel sample is placed inside the ring. The thickness of the sample in the neutron beam is therefore also 1.2 mm. Using a neutron wavelength of 6 $\mathring{A}$ and a collimation length of 12.8 m, a total q-range of $2.6 \cdot 10^{-3}$ to $4.7 \cdot 10^{-1}$ $\mathring{A}$ was covered in one configuration. Data reduction was done using Grasp.[15] The raw intensity data were corrected for background scattering and weighted by the transmission of the sample. The electronic

background noise was attenuated using a sintered $^{10}B_4C$ sample and absolute scaling was obtained by measuring the direct beam on the detector. Finally, the 2D data were radially averaged using Grasp.

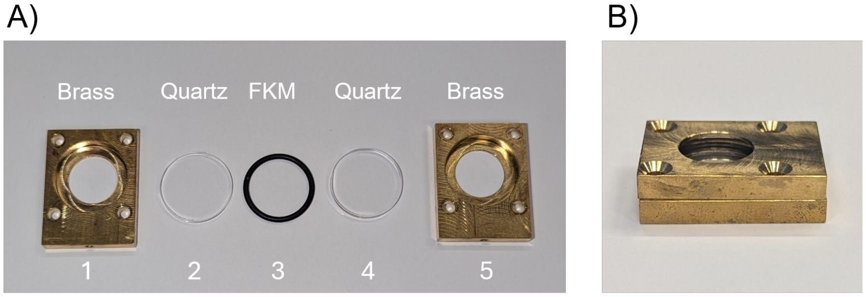

Figure 19: Photos of sandwich cell for gel samples. A) Materials of the individual cell components. The number indicates the order in which the compartments are stacked in the final cell. B) Side view of the stacked sandwich cell.

To determine the incoherent background scattering intensity, bkg, the generalized Porod law was fitted to the data for $q > 0.1\,\AA^{-1}$:[16]

$$I(q) = A \cdot q^{-m} + \mathrm{bkg}\,, \tag{19}$$

where A is a constant and m is the Porod exponent ($m = 4$ for sharp surfaces). The data shown in the main text represent the measured intensities with subtracted incoherent background scattering.

Hammouda Model Analysis

A model that is frequently used to describe the scattering from hydrogels or semidilute polymer solutions with large-scale inhomogeneities, is the Hammouda model:[17]

$$I(q) = \frac{A}{q^n} + \frac{B}{1 + (\xi q)^m}\,, \tag{20}$$

where A and B are constants. The first term is the Porod scattering of the large scale imhomogeneities, characterized by the Porod exponent, n. The second term describes the Ornstein-Zernike scattering of the polymer chains ($m = 2$ for ideal Gaussian behavior). ξ is the correlation length. The SANS data of the modified tetra-PEG samples was fitted with the Hammouda model. The results are shown in Figure 20. The model fits the data

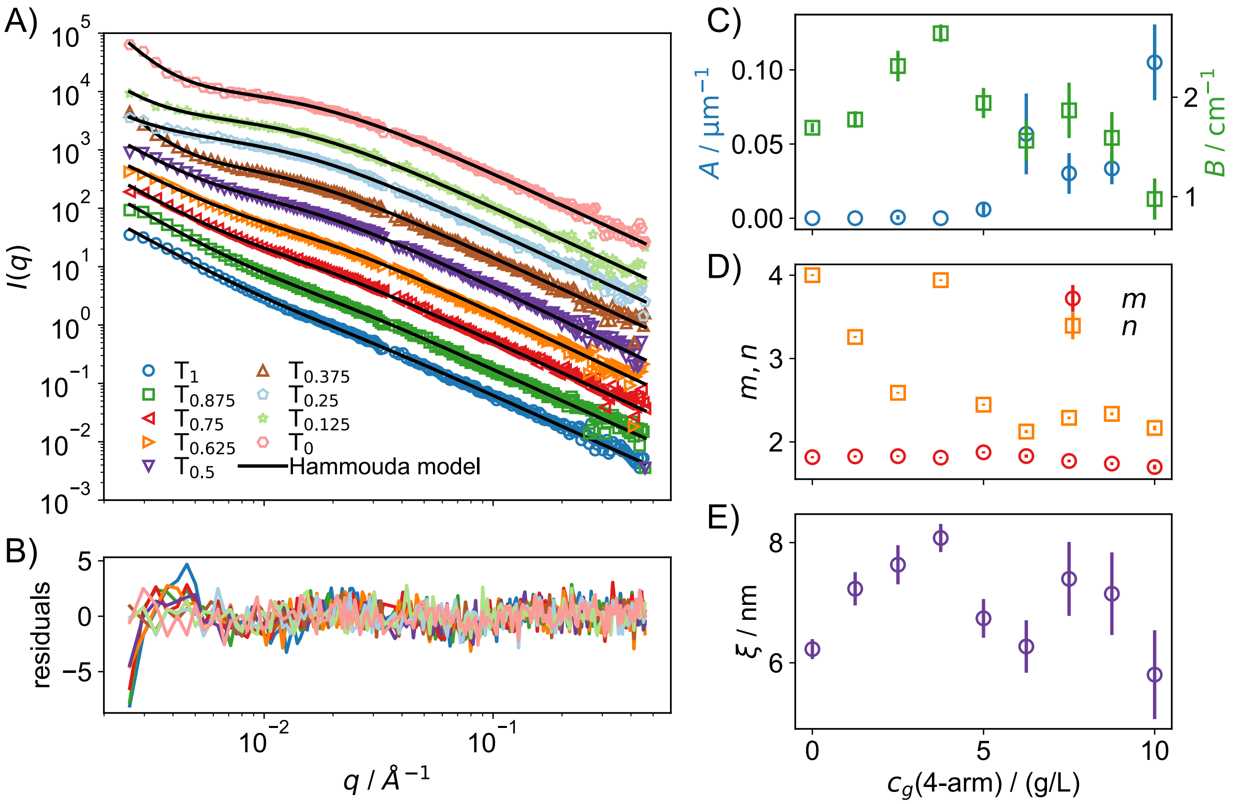

Figure 20: Results of the Hammouda model fits. A) Modified tetra-PEG SANS spectra, fitted with the Hammouda model. The T_1 curve is in cm^{-1}. The remaining curves were multiplied with 3^i, where $i = 1, 2, 3, \ldots$, to improve readability. B) Fit residuals. C)-E) Results for fit parameters.

very across all samples and q values. However, looking at the fit parameter results, no clear trends of the parameters are visible as a function of the cross-linker concentration. The SANS spectra have very few distinct features. This is particularly visible for the stiffer hydrogels, which almost look like straight lines in the double logarithmic plot. This indicates that the samples have the same fractal dimension over the entire length scaled probed by the SANS experiment. Fitting such a featureless curve with a model containing five parameters leads to

large inaccuracies. To draw meaningful information from this fit, one would have to fix some of the parameters. However, other than m, which is equal to the Porod exponent determined for the background subtraction, none of the parameters are known *a priori*. For this reason, we have decided to focus instead on the difference between data and Ornstein-Zernike term, which should be equal across all samples, as explained in detail in the main text.

References

(1) Fujiyabu, T.; Li, X.; Shibayama, M.; Chung, U.-i.; Sakai, T. Permeation of Water through Hydrogels with Controlled Network Structure. *Macromolecules* **2017**, *50*, 9411–9416, DOI: 10.1021/acs.macromol.7b01807.

(2) Fujiyabu, T.; Li, X.; Chung, U.-i.; Sakai, T. Diffusion Behavior of Water Molecules in Hydrogels with Controlled Network Structure. *Macromolecules* **2019**, *52*, 1923–1929, DOI: 10.1021/acs.macromol.8b02488.

(3) Northrop, B. H.; Frayne, S. H.; Choudhary, U. Thiol–maleimide "click" chemistry: evaluating the influence of solvent, initiator, and thiol on the reaction mechanism, kinetics, and selectivity. *Polymer Chemistry* **2015**, *6*, 3415–3430, DOI: 10.1039/C5PY00168D.

(4) Matsunaga, T.; Sakai, T.; Akagi, Y.; Chung, U.-i.; Shibayama, M. Structure Characterization of Tetra-PEG Gel by Small-Angle Neutron Scattering. *Macromolecules* **2009**, *42*, 1344–1351, DOI: 10.1021/ma802280n.

(5) Miller, D. R.; Macosko, C. W. A New Derivation of Post Gel Properties of Network Polymers. *Macromolecules* **1976**, *9*, 206–211, DOI: 10.1021/ma60050a004.

(6) Lutolf, M. P.; Hubbell, J. A. Synthesis and Physicochemical Characterization of End-Linked Poly(ethylene glycol)- *co* -peptide Hydrogels Formed by Michael-Type Addition. *Biomacromolecules* **2003**, *4*, 713–722, DOI: 10.1021/bm025744e.

(7) Gordon, M.; Scantlebury, G. R. Statistical kinetics of polyesterification of adipic acid with pentaerythritol or trimethylol ethane. *Journal of the Chemical Society B: Physical Organic* **1967**, 1, DOI: 10.1039/j29670000001.

(8) Mason, T. G.; Weitz, D. A. Optical Measurements of Frequency-Dependent Linear Viscoelastic Moduli of Complex Fluids. *Physical Review Letters* **1995**, *74*, 1250–1253, DOI: 10.1103/PhysRevLett.74.1250.

(9) Mason, T.; Gang, H.; Weitz, D. Rheology of complex fluids measured by dynamic light scattering. *Journal of Molecular Structure* **1996**, *383*, 81–90, DOI: 10.1016/S0022-2860(96)09272-1.

(10) Mason, T. G. Estimating the viscoelastic moduli of complex fluids using the generalized Stokes-Einstein equation. *Rheologica Acta* **2000**, *39*, 371–378, DOI: 10.1007/s003970000094.

(11) Durran, D. R. In *Numerical Methods for Wave Equations in Geophysical Fluid Dynamics*; Marsden, J. E., Sirovich, L., Golubitsky, M., Jäger, W., Eds.; Texts in Applied Mathematics; Springer New York: New York, NY, 1999; Vol. 32; DOI: 10.1007/978-1-4757-3081-4.

(12) Quarteroni, A.; Sacco, R.; Saleri, F. *Numerical Mathematics*; Texts in Applied Mathematics; Springer New York: New York, NY, 2007; Vol. 37; DOI: 10.1007/b98885.

(13) Fornberg, B. Generation of finite difference formulas on arbitrarily spaced grids. *Mathematics of Computation* **1988**, *51*, 699–706, DOI: 10.1090/S0025-5718-1988-0935077-0.

(14) Galantini, L.; Ariodante, L.; Bains, A.; Edler, K. J.; Gradzielski, M.; Matsarskaia, O.; Nylander, T.; Omasta, T.; Prévost, S.; Schillén, K. Bile Salt-Driven Innovations: Exploring Deep Eutectic Solvents for Sustainable Solutions. 2024; https://doi.org/10.5291/ILL-DATA.9-10-1840.

(15) Dewhurst, C. D. Graphical reduction and analysis small-angle neutron scattering program: *GRASP*. *Journal of Applied Crystallography* **2023**, *56*, 1595–1609, DOI: 10.1107/S1600576723007379.

(16) Porod, G. Die Röntgenkleinwinkelstreuung von dichtgepackten kolloiden Systemen: I. Teil. *Kolloid-Zeitschrift* **1951**, *124*, 83–114, DOI: 10.1007/BF01512792.

(17) Hammouda, B.; Ho, D. L.; Kline, S. Insight into Clustering in Poly(ethylene oxide) Solutions. *Macromolecules* **2004**, *37*, 6932–6937, DOI: 10.1021/ma049623d.
